# Current Analogues of Future Climate Indicate the Likely Response of a Sensitive Montane Tropical Avifauna to a Warming World

**DOI:** 10.1371/journal.pone.0069393

**Published:** 2013-07-31

**Authors:** Alexander S. Anderson, Collin J. Storlie, Luke P. Shoo, Richard G. Pearson, Stephen E. Williams

**Affiliations:** 1 Centre for Tropical Biodiversity and Climate Change, School of Marine and Tropical Biology, James Cook University, Townsville, Queensland, Australia; 2 School of Biological Sciences, The University of Queensland, St. Lucia, Queensland, Australia; 3 School of Marine and Tropical Biology, James Cook University, Townsville, Queensland, Australia; University of Pretoria, South Africa

## Abstract

Among birds, tropical montane species are likely to be among the most vulnerable to climate change, yet little is known about how climate drives their distributions, nor how to predict their likely responses to temperature increases. Correlative models of species’ environmental niches have been widely used to predict changes in distribution, but direct tests of the relationship between key variables, such as temperature, and species’ actual distributions are few. In the absence of historical data with which to compare observations and detect shifts, space-for-time substitutions, where warmer locations are used as analogues of future conditions, offer an opportunity to test for species’ responses to climate. We collected density data for rainforest birds across elevational gradients in northern and southern subregions within the Australian Wet Tropics (AWT). Using environmental optima calculated from elevational density profiles, we detected a significant elevational difference between the two regions in ten of 26 species. More species showed a positive (19 spp.) than negative (7 spp.) displacement, with a median difference of ∼80.6 m across the species analysed that is concordant with that expected due to latitudinal temperature differences (∼75.5 m). Models of temperature gradients derived from broad-scale climate surfaces showed comparable performance to those based on in-situ measurements, suggesting the former is sufficient for modeling impacts. These findings not only confirm temperature as an important factor driving elevational distributions of these species, but also suggest species will shift upslope to track their preferred environmental conditions. Our approach uses optima calculated from elevational density profiles, offering a data-efficient alternative to distribution limits for gauging climate constraints, and is sensitive enough to detect distribution shifts in this avifauna in response to temperature changes of as little as 0.4 degrees. We foresee important applications in the urgent task of detecting and monitoring impacts of climate change on montane tropical biodiversity.

## Introduction

Evidence for warming of the global climate system is unequivocal, with widespread rises in air and sea temperatures likely driven by anthropogenic increases of atmospheric CO_2_ to concentrations well above pre-industrial levels [1]. Extinctions due to the rapid rate of current change [2] may profoundly impact global patterns of biodiversity [3]. While the magnitude of measured temperature changes has been greater in high latitudes [1], steep gradients and narrow thermal tolerances may make tropical montane ecosystems particularly vulnerable [4]–[6]. As a result, climate change represents perhaps the most significant threat to tropical montane biodiversity [7], [8], with substantial losses to extinction expected in coming centuries if warming remains unchecked [2], [9]. Research efforts to date have often focused on temperate montane systems [10]–[12], leaving a knowledge gap in both predicted and documented impacts in their tropical equivalents [13]. There is thus an urgent need to validate projected impacts of climate change on montane tropical bird species [14].

Species distribution modelling is widely used to predict potential impacts of climate change on flora and fauna. However, such models rely on correlations that implicitly assume causal relationships between species distributions and environmental variables. Thus independent tests of the assumption that climate factors drive the distributions of species are urgently needed [15]. Hindcasting and a substitution of space for time are two approaches that can be used for this purpose [3]. Where historical data are available, hindcasting has already identified numerous cases of up-slope shifts in response to rapid temperature increases in the latter part of last century [10]–[13], [16]. However, historical data are lacking for many ecosystems, and particularly in species-rich but data-poor tropical systems. In such situations, space-for-time substitutions, where warmer locations are used as analogues of future conditions, may serve as a crucial tool for evaluating the assumptions of species distribution models in the context of climate change [17]. A second important challenge lies in how to measure distribution differences [18]. Efforts to quantify distribution differences in both space and time have often emphasised detecting change at the margins of species distributions [13], [19], [20]. However, the low occupancy and abundance often observed at distribution margins can hinder the accurate definition of these limits, as detection is sensitive to sampling effort [18]. Consequently, analytical approaches are increasingly being directed toward measures of central tendency (or optima) as they use more of the available data and are less affected by sampling bias [11].

Defining species distributions by their environmental optima is not without its own complications, however, as species’ responses to environmental gradients can take a variety of forms [21]. As a result it may be inaccurate to assume that the response of a particular species to an environmental gradient (e.g. temperature or elevation) will have a single clearly defined optimum. For the purposes of detecting range-shifts, however, the problem can be simplified by concentrating on those species for which a response model with a clearly defined optimum is the most appropriate. The Gaussian response is one such model, often applied to species distributions across environmental gradients, for which it is possible to identify the optimum with confidence intervals [22]. This approach allows statistical comparison of the location of density optima, and has been used to discern elevational range shifts over time [11], [23]. There is considerable scope to extend this same analytical approach to evaluate contemporary constraints of environment on distribution by examining elevational differences between density optima along secondary spatial environmental gradients such as latitude. By employing warmer locations as analogues of future climate, such a “space-for-time” substitution can be used to directly examine the evidence for a causative relationship between species environmental tolerances and their spatial distribution, and hence infer the tendency for species’ distributions to track environmental change without the need for historical data [3]. The selection of an appropriate environmental gradient against which to measure shifts is also an important consideration. Climate predictions suggest that maximum and minimum temperatures are increasing more rapidly than mean values in some regions [24]. Some species may also be particularly vulnerable to extremes of heat and cold [25], and hence may track maxima or minima more closely than average values. Changes in mean annual temperatures may therefore be a poor predictor of species distribution changes in some cases, necessitating the inclusion of other parameters of thermal gradients.

Here we use an extensive data set on the density of rainforest birds across elevational gradients in north-eastern Australia to identify species for which temperature is likely a major driver of their distributions across elevation. We then test whether temperature differences provide a parsimonious explanation for variation in the positioning of species along spatial gradients. The region has been identified as an Important Bird Area [26], highlighting its contribution to Australia’s avifaunal diversity [27]. Previous studies have also predicted a high level of vulnerability to climate change among upland endemic rainforest species in the region [9], [28], [29]. Here we examine evidence for upslope displacement of the elevational optima of populations at lower (warmer) latitudes, consistent with expectations based on the gradient of temperature across elevation, an important assumption of the above climate change predictions. First, we use a hierarchical modeling approach [30] to select species whose density response along elevational gradients can be well approximated by a unimodal (Gaussian) curve, as these species are not only likely to be sensitive to climate change, and therefore of conservation concern, but in addition their response may also be more readily measured. We then characterise the selected subset of species by their environmental optima across the elevational gradient, applying simple logistic regression to estimate the location of their peak density, with confidence intervals [22]. Finally, we quantify directional differences in elevations of density optima between latitudes and discuss the implications of our findings for predicting impacts of anthropogenic climate change on biological communities, and for monitoring of the resulting temporal range shifts.

## Materials and Methods

### Study area

**Figure 1:**
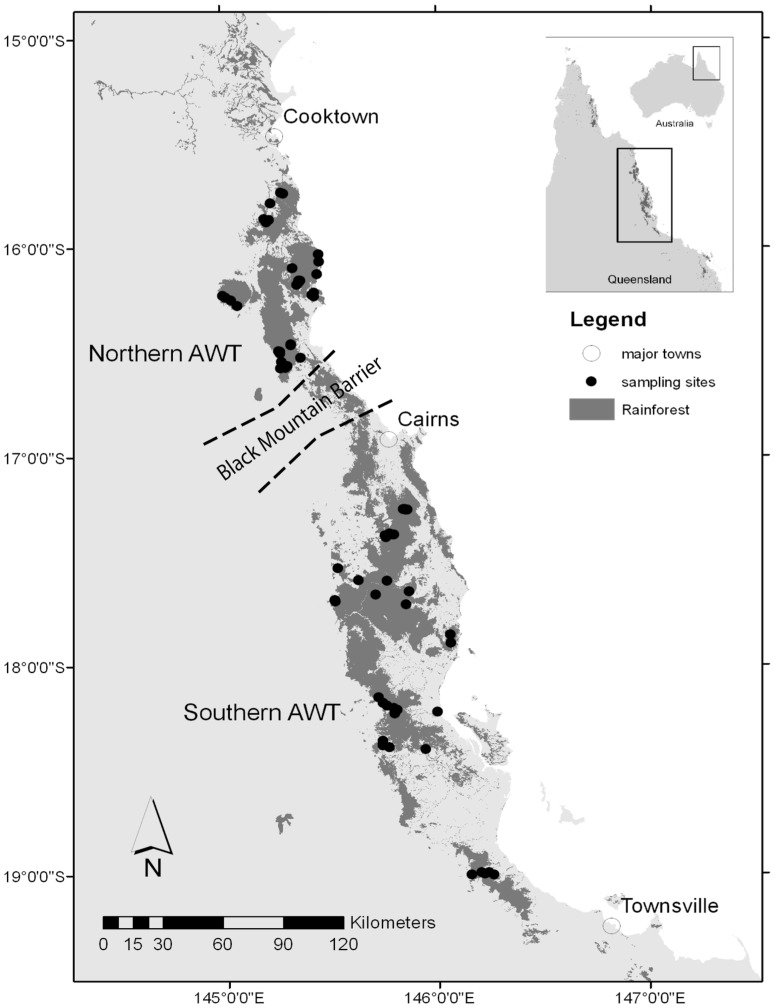
Rainforests sampling areas within the study region. Areas dominated by rainforest vegetation are shaded in dark grey. Dotted lines indicate a major biogeographic barrier (the Black Mountain barrier, see text) separating the northern and southern AWT regions compared in this study.

We analyse bird density data collected in the Australian Wet Tropics (AWT) Bioregion between −15°45′32.69′′S 145° 1′53.87′′E and −19°18′0.65′′S 146° 9′41.17′′E). Rainforests in this part of north-eastern Queensland are associated with coastal ranges of the Great Dividing Range and adjacent lowlands, giving a substantial elevational range between sea level and 1645 m ASL. The structure and floristics of rainforest across this gradient varies from complex mesophyll vine forest in the coastal lowlands to notophyll vine forest and microphyll fern thicket on high peaks and plateaus, although the majority of data in this study were collected in simple to complex notophyll vine forests [31]. Sampling here is focused on two discrete sections of the AWT, separated by the Black Mountain barrier [32], [33]. These are: the northern AWT: between Cairns (∼ −17°S) and Cooktown (∼ −15.5°S), and the southern AWT: south of Cairns to about −19.5°S near Townsville (Fig. 1). Across this biogeographic barrier there is little difference in the avifauna, though several species are split into distinct lineages [34]. The climate of the region is characterised by warm average temperatures and high rainfall concentrated in the summer wet season (October to May). Upland forests experience higher rainfall and lower temperatures than lowland forests, and seasonality of rainfall decreases from north to south and from lowlands to uplands, while seasonality of temperature follows the reverse trend. The northern and southern AWT thus represent two contrasting thermal gradients in which to compare the elevational responses of populations of rainforest birds, with gradients in the northern AWT being shifted upslope by the effect of latitude and the adiabatic lapse rate on temperature.

### Bird density estimation

Bird survey data from a total of 944 surveys across 97 sites were employed in this analysis. Rainforest birds were surveyed at arrays of 6 points located at 200 m intervals along a 1-km transect at each study site. Sites were distributed at 200-m intervals across the elevational gradient from sea level to the summits of the highest peaks in northern and southern parts of the region (1320 and 1645 m, respectively). Sampling methodology followed Williams et al. [35], with surveys conducted between dawn and 9:30 am, and consisting of 30-minute, 150-m fixed-width transects. Each site was surveyed an average of 9 times to allow the estimation of mean abundance of each species present at the site across all surveys at that site. Surveys were conducted during both the wet (summer) and dry (winter) seasons. We wished to obtain the best measure of species optima, and so used distance sampling on a subset of surveys covering the entire gradient, to control for the effect of detectability differences which may bias estimates of relative abundance. In these surveys, the perpendicular distance of all individuals from the survey transect was estimated with aid of an Opti-Logic LH400 Laser Range finder (http://www.opti-logic.com/lh_series.htm) and analysed in the software program Distance [36] to derive a detection function for each species that characterised the decay of detectability with distance from the transect. This detection function was then used to estimate the ‘Effective Strip Width’ (ESW, defined in distance sampling as the transect half-width at which the total count over the area L×(2×ESW) would be on average equal to the observed count (where L  =  survey length) [36]) for each species. ESWs for each species and site were then used as the multiplier in calibrations of mean relative abundances to give an absolute density estimate for each transect. Sampling was sufficient to calculate density directly for common species at a site using the Distance software [36]. For less common species, however, records within a site were often too few to accurately fit a detection function, in which case data were pooled among nearby sites until samples size was sufficient, and the resulting broader calibration applied instead. As we are interested in a temperature response, and temperature increases have already begun to be felt, only data from surveys between January 2000 and June 2010 are included in this analysis, providing a recent snapshot of the patterns of rainforest bird elevational density responses across the environmental gradient.

### Expected elevational shifts

We characterised the thermal gradient across elevation in each subregion in terms of mean annual (MAT), minimum (T_min_) and maximum (T_max_) temperatures. We used temperature data from modeled climate surfaces with bioCLIM in the ANUCLIM 5.1 software [37] which uses a splined model of the relationship between temperature, measured at standard meteorological weather stations, combined with an 80 m resolution DEM (resampled from GEODATA 9 Second DEM Version2; Geosciences Australia, http://www.go.gov.au/). We also characterised the actual thermal gradient likely to be experienced by the forest avifauna using under-canopy microclimate data collected with a thermal logger array across the entire elevational range of this study [38]. Briefly, temperature data were collected from a network of weather stations (n = 27) distributed throughout the latitudinal and altitudinal gradients of the study region. Weather stations were positioned underneath dense rainforest canopy at a height of approximately 1.3 metres above the ground. Measurements of temperature were recorded every 30 minutes using a HOBO 8-Bit Temperature Sensor (http://www.microdaq.com/occ/hws/micro_station.php) or an iButton thermochron (http://www.maxim-ic.com/products/ibutton/) from November 2006–June 2009. Additional rainforest sites (n = 14) were also monitored intermittently over the period June 2004–June 2009 using thermochron temperature loggers sampling at the same height and time interval. This empirical dataset was supplemented with climate data provided by the Australian Bureau of Meteorology (BoM, http://www.bom.gov.au) to generate a splined surface of microclimate data, (“accuCLIM”). For a detailed description of these methods see [38].

Subsequent statistical analyses were all conducted in the “R” framework for statistical analysis version 2.13.1 [39]. Elevational temperature profiles were generated by querying the accuCLIM [38] and bioCLIM MAT, T_min_ and T_max_ temperature layers at a random subset of 150 points from the standard sampling arrays used for bird surveying in the northern and southern AWT, (to give equal sample sizes). We then characterised each of these using simple linear models from which we generated predictions of the temperatures likely to be experienced by birds at any given elevation in the north or south. These predictions were later used to test the performance of each temperature parameter as a predictor of observed latitudinal differences in the elevations of bird density optima.

### Density profile modelling

Calibrated density information was available for 115 species. We excluded any species that lacked sufficient data to accurately model a density response (set at <10 survey points) across the elevational gradient in either the southern or northern AWT. Importantly, to select species likely to be sensitive to changes in thermal conditions, we further limited analysis to those species for which the temperature response for all data combined approximated a unimodal curve, showing a clear optimum at which estimated density reaches a maximum. We used the Huisman-Olff-Fresco (HOF) hierarchical modeling approach [30], implemented in the R package “BiodiversityR” [40] to select only those species whose density profiles are best characterised by a unimodal distribution. In the HOF analysis, species density profiles were compared to flat, monotonic, plateau, Gaussian, and skewed distributions. The most appropriate model was selected using Aikake’s Information Criterion (AIC). Only species displaying a unimodal (Gaussian or skewed) response were included in further analyses. While skewed abundance distributions may be relatively common across natural gradients [30], [41], symmetrical distributions are widely used to approximate abundance responses in community ecology, and simplify the process of identification of optima and confidence intervals. Finally we tested for the presence of systematic taxonomic or ecological patterns in the distribution of density profile responses using Chi-squared tests (data not shown).

### Observed elevational differences

For the subset of species selected using the HOF analysis, the elevations of optimal density in the southern and northern AWT were then identified using the approach of Oksanen et al. [21]. This approach fits a Gaussian curve to the patterns of species’ mean density across an environmental gradient using simple logistic regression. Defining the maximum density value as the peak of the unimodal curve, we then calculated confidence intervals around the optimum using a Fieller likelihood method implemented by Oksanen and Minchin [21]. As densities were generally low, and data often included zeros (absences), we expressed density as a proportion of maximum density for each species, and assumed a binomial error distribution, though selection of alternative error distributions (poisson and quasi-poisson) did not substantially alter the resulting model assignments. We then compared the elevational optima of each species between its northern and southern AWT populations. We assessed the significance of the observed elevational differences based on the overlap or non-overlap of southern upper, and northern lower 84% confidence intervals, suggested in [42] as more appropriate for hypothesis testing than 95% intervals. While commonly quoted, comparison of 95% confidence intervals increase the likelihood of type 1 error (here, falsely concluding differences in optimum elevation to be non-significant based on overlapping CIs). We then tested for the presence of systematic ecological or taxonomic patterns in the distribution of elevational optimum differences among species responses using Chi-squared tests.

### Comparison with predicted differences

Pooling these estimated latitudinal differences across species, we used Wilcoxon–Mann–Whitney one-sample rank-sum tests to compare the median of the observed elevational optima differences between the northern and southern AWT to that predicted by the elevational gradient of each temperature parameter. As temperature gradient slopes for T_ max_ and T_ min_ were not parallel, we also examined the Root Mean Square Errors associated with a regression of the observed and predicted values for each species/climate parameter combination, which preserves the information contained in each species response. Using linear models of the relationship between climate and elevation (described above), we first identified each species’ optimum *temperature* based on the *elevation* of its density optimum and the local temperature gradients in the southern AWT. Using a linear model of the same climate/elevation relationships in the northern AWT, we then predicted the northern *elevation* that corresponded to that species’ southern optimum *temperature*. When repeated for each parameter, comparison with observed elevation of the northern density optima indicated the best predictors of elevational density response.

## Results

### Expected elevational differences

**Figure 2:**
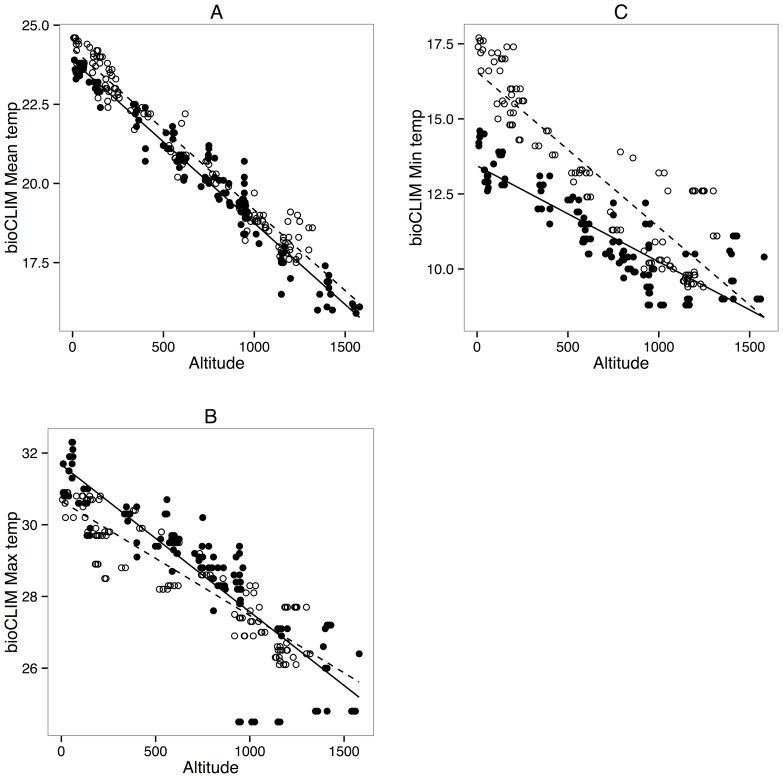
Relationships between elevation and temperature parameters for the study region. A: Mean Annual Temperature (MAT), B: Maximum Temperature of the Warmest Period (T_max_) and C: Minimum Temperature of the Coolest Period (T_min_). Data were interpolated from bioCLIM, and sites in the southern AWT (filled circles) and northern AWT (unfilled circles) are indicated. The solid lines are simple linear models of the effect of elevation on temperature for each parameter, with the trend for southern sites shown by a solid line, that for northern sites with a dashed line. Corresponding data for accuCLIM climate surfaces (see text) are shown in figure S1.

Linear models of elevational temperature lapses between subregions for MAT, T_ max_ and T_ min_ estimated from bioCLIM showed a clear elevational and latitudinal lapse pattern: MAT decreased by ∼5.1° per 1000 m elevation in both regions, but were an average of 0.41° warmer (SE 0.04°) in the north based on the difference in intercepts of regressions between temperature and elevation (Fig. 2A). This increase in temperature translated to a 75.57 m (SE 7.61 m) upward displacement in the thermal gradient between the two regions, and provides an estimate of the expected increase in optimum elevation required for species to experience the same average temperature regime across their density profiles in the southern and northern subregions. In contrast, regressions for T_ max_ (Fig. 2B) and T_ min_ (Fig. 2C) were not parallel, showing significant subregion interaction terms, (T_max_ multiple r^2^  = 0.79, subregion term: t = −3.24, P = <0.013, T_min_ multiple r^2^  = 0.84, subregion term: t = 9.507, P = <0.001). These patterns result in an estimated difference of −0.38°/−81.18 m (SE 0.04°/16.64 m) between southern and northern regions for T_max_ (so that northern sites experience cooler maximum temperatures) and +1.77°/+403.57 m (SE 0.11°/22.44 m) for T_min_. There was little qualitative difference between these and the elevational temperature gradients estimated in the accuCLIM data set [63] (Fig. S1) but the accuCLIM MAT temperature differential was slightly smaller, yielding temperatures on average 0.35° (SE 0.07°) warmer, translating to a 54.89 m (SE 11.06 m) elevational difference. In contrast, estimated differences for accuCLIM T_max_ (−1.47°/−171.68, SE 0.04°/16.64 m) and T_min_ (+2.09°/+487.58 m, SE 0.14°/30.56 m) were larger.

### Density profile modelling

A total of 154 species were detected during this study, of which 108 (70%) had sufficient data to accurately estimate their densities across elevation. Of these, 80 (74%) had sufficient detections and abundance to be tractable using the HOF Hierarchical model testing approach [30]. This identified 47 species as exhibiting a unimodal temperature response (a Gaussian (type IV, 13 spp.), or skewed response (type V, 34 spp.)), and a further 9 with a monotonic (type II) and 23 a plateau (type III) response (summarised in Table 1, and shown in full in supplementary material: Table S1, and Figs. S2.1–S1.80). Importantly, in most taxa for which a skewed model returned a higher AIC score, the skewed model tended to generate similar estimates of the location of the optimum to the simple Gaussian response (e.g. Brown Gerygone (*Gerygone mouki*) Fig. S2.11 and Bridled Honeyeater (*Lichenostomus frenatus*) Fig. S2.12). As subsequent model testing methods apply only to unimodal distributions, species with a plateau (type III) response (e.g. Grey-headed Robin (*Heteromyias albispecularis*) Fig. S2.32), or monotonic positive (type II) response (e.g. Double-eyed -Fig-parrot (*Cyclopsitta diopthalma*) Fig. S2.21), were excluded. It is important to note, however, that these species may well have a unimodal temperature response, but one that is truncated by the limits of the available temperature gradient (see discussion). Response types were distributed across the range of taxonomic, phylogeographic and ecological groups examined, and we found no significant trend in the distribution of unimodal responses across these factors.

**Table 1:** Number and type of species’ elevational responses.

Model Number	Model name	Count of species
**I.**	Flat	2
**II.**	Monotonic	11
**III.**	Plateau	18
**IV.**	Gaussian	18
**V.**	Skewed	28

The number of flat, plateau, monotonic positive, negative, Gaussian and skewed response detected using the Huisman-Olff-Fresco [30] approach (see text for details).

### Observed elevational differences

**Figure 3:**
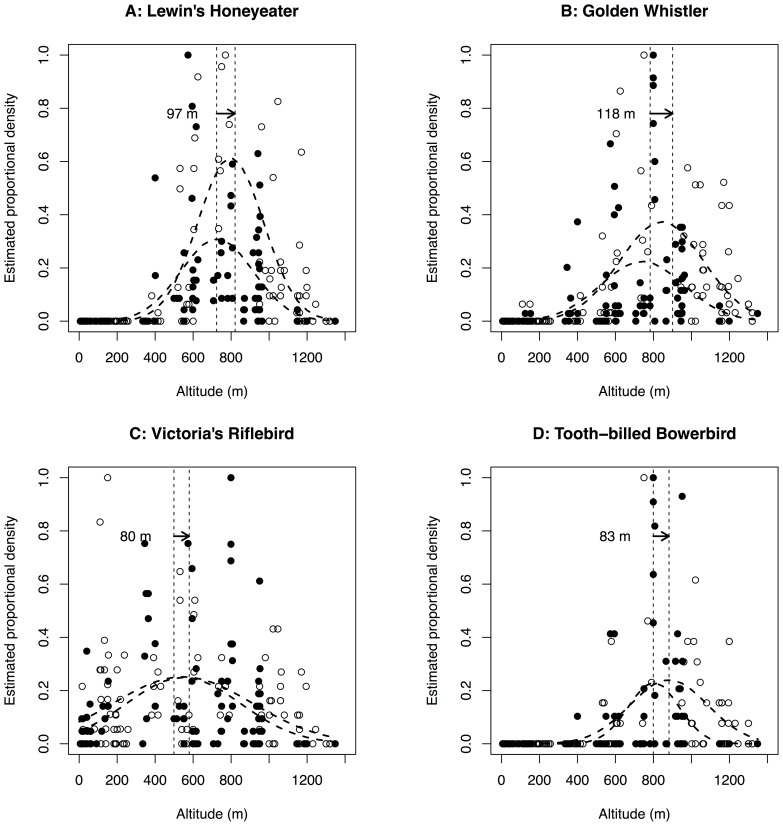
Elevational density profiles. Shown are example plots for 4 of the 10 species exhibiting a significant difference between the elevation of density optima between southern (filled circles) and northern (unfilled circles) AWT populations according to 84% Confidence Intervals (see text for explanation of this choice). Data are proportional estimated densities corrected for detectability at each sampling point. The vertical lines mark the estimated elevations of density optima in the two regions. Arrows and their labels indicate the direction and magnitude of the estimated elevational shift in each case. See table 2 for species’ scientific names.

Of the 47 species exhibiting a unimodal (Gaussian or skewed) temperature response, we considered 26 (55%) to also be amenable to the approach used in Oksanen et al. [21] for calculating the location and confidence intervals of the density optimum, having both sufficient sampling coverage (occupancy at 3 or more sites) and an optimum at least 100 metres from the bounds of the elevational domain in this study (inclusion of species with optima closer to the upper and lower limits of the gradient creates problems for model fitting with this approach [11], [21]). Table 2 shows the results of the Gaussian optimum calculations for these species, for which a Gaussian model explained a mean of 34% of deviation in both northern and southern populations (range 3.6% to 71.7%). Across the taxa identified using the above criteria, a further 19 (73%) species showed a positive elevational difference. This difference was indicated as significant in ten species by non-overlap of 84% confidence intervals between the southern and northern optima. Superimposing southern and northern elevational density profiles for four of these (Fig. 3) illustrates the nature of these differences (remaining taxa shown in Supplementary material, Figs. S3.1–S2.14). As with the response types, we found no significant trend in the distribution of elevational optima differences among taxonomic, endemicity or rainforest specialization species groupings (data not shown).

**Table 2:** Estimated elevations of species’ density optima.

		Southern AWT					Northern AWT					
	Common name	Optimum elevation (m)	lower 84% CI(Fieller)	upper 84% CI (Fieller)	%deviance explained	# Sites	Optimum elevation (m)	lower 84% CI (Fieller)	upper 84% CI (Fieller)	%deviance explained	# Sites	Estimated altitudinal shift (m)
	COLUMBIFORMES											
1	**Brown Cuckoo-Dove (** ***Macropygia amboinensis*** **)^S^**	**556.42**	**500.11**	**626.65**	**4.58**	**71**	**745.43**	**704.53**	**794.6**	**13.62**	**54**	*189.01*
2	Topknot Pigeon (*Lopholaimus antarcticus*)^S^	755.82	703.13	807.57	28.08	14	659.13	480.95	894.68	22.61	13	−96.69
	PSITTACIFORMES											
**3**	Sulphur-crested Cockatoo (*Cacatua galerita*)	535.7	478.53	602.32	9.37	70	491.11	450.25	525.77	21.21	54	−44.59
	CUCULIFORMES											
4	Shining Bronze-Cuckoo (*Chalcites lucidus*)	725.89	668.78	798.89	20.97	17	798.29	784.34	811.87	55.84	17	*72.41*
	CORACIIFORMES											
**5**	Rainbow Bee-eater (*Merops ornatus*)	348.72	309.52	388.31	27.92	16	429.61	371.15	484.67	25.33	24	*80.89*
	PASSERIFORMES											
6	White-throated Treecreeper (*Cormobates leucophaea*)	951.07	903.05	1017.74	55.09	59	805.61	790.33	821.01	65.81	36	−145.45
**7**	Brown Gerygone (*Gerygone mouki*)^S^	635.57	599.21	679.4	41.29	83	582.04	523.84	644.98	36.76	39	−53.53
**8**	**Fernwren (** ***Oreoscopus gutturalis*** **)^E,S^**	**797.9**	**754.45**	**857.1**	**44.25**	**53**	**978.51**	**893.4**	**1141.81**	**39.32**	**52**	*180.62*
9	Mountain Thornbill (*Acanthiza katherina*) ^E,S^	1193.13	1058.76	2012.18	50.1	27	1147.9	1057.8	1342.12	67.65	38	−45.22
**10**	Yellow-throated Scrubwren (*Sericornis citreogularis*) ^E,S^	766.26	717.21	828.29	42.98	36	795.99	727.65	879.9	42.88	38	*29.73*
**11**	Bridled Honeyeater (*Lichenostomus frenatus*) ^E,S^	872.82	786.15	1055.37	32.76	45	1144.35	965.01	1951.55	33.5	49	*271.53*
12	Eastern Spinebill (*Acanthorhynchus tenuirostris*)	932.17	815.44	1260.22	26.38	32	945.68	915.27	981.43	55.75	34	*13.51*
**13**	**Lewin's Honeyeater (** ***Meliphaga lewinii*** **)**	**723.76**	**712.24**	**735.46**	**67.25**	**62**	**820.84**	**810.19**	**831.45**	**71.7**	**34**	*97.08*
**14**	**Scarlet Honeyeater (** ***Myzomela sanguinolenta*** **)**	**130.17**	−**267.66**	**227.96**	**24.29**	**13**	**654.74**	**638.87**	**670.66**	**58.62**	**10**	*524.56*
**15**	**Chowchilla (** ***Orthonyx spaldingii*** **)^E,S^**	**721.51**	**671.98**	**791.43**	**25.97**	**74**	**894.38**	**862.27**	**933.41**	**45.27**	**52**	*172.87*
**16**	**Eastern Whipbird (** ***Psophodes olivaceus*** **)**	**576.54**	**504.67**	**682.32**	**3.56**	**91**	**909.67**	**889.55**	**932.19**	**58.48**	**49**	*333.12*
17	Bowers Shrike-Thrush (*Colluricincla boweri*)^E,S^	886.75	779.13	1148.02	28.25	45	897.83	878.63	918.44	61.54	38	*11.08*
**18**	**Golden Whistler (** ***Pachycephala pectoralis*** **)**	**782.59**	**747.19**	**827.37**	**49.59**	**64**	**900.9**	**862.67**	**948.43**	**54.38**	**47**	*118.3*
19	Grey Fantail (*Rhipidura albiscapa*)	698.33	606.43	904.9	15.03	69	756.21	699.47	830.69	21.45	62	*57.88*
20	Spectacled Monarch (*Symposiarchus trivirgatus*)^S^	113.6	−960.32	278.8	25.16	84	358.78	51.12	472.19	21.3	68	*245.18*
**21**	White-eared Monarch (*Carterornis leucotis*)^S^	403.93	348	453.38	15.09	20	274.53	−1215.35	444.97	8.99	16	−129.4
**22**	**Victoria's Riflebird (** ***Ptiloris victoriae*** **)^E, S^**	**498.89**	**460.16**	**537.84**	**8.85**	**75**	**579.32**	**554.55**	**603.08**	**24.08**	**61**	*80.43*
**23**	Satin Bowerbird (*Ptilonorhynchus violaceus*)^S^	887.38	809.23	1117.58	26.94	16	865.77	662.6	2324.39	16.04	7	−21.61
24	Spotted Catbird (*Ailuroedus melanotis*)^S^	550.98	506.72	602.58	15.08	82	642.76	601.6	684.24	17.71	71	*91.78*
25	**Tooth-billed Bowerbird (** ***Scenopoeetes dentirostris*** **)^E,S^**	**799.01**	**762.65**	**849.06**	**35.7**	**27**	**881.9**	**851.07**	**915.79**	**47.74**	**28**	*82.89*
26	**Silvereye (** ***Zosterops lateralis*** **)**	**262.54**	−**61.84**	**360**	**19.6**	**73**	**525.41**	**472.2**	**575.61**	**39.8**	**61**	*262.87*

Elevations of density optima for southern and northern AWT populations of the 26 rainforest bird species identified as having a unimodal (Gaussian or skewed) temperature response, with optima at least 100 m from the gradient limits, which could be estimated using the approach in Oksanen et al. [22]. Species are shown in alphabetical order, with their optimum elevations and upper and lower 84% confidence intervals, as well as the estimated south/north difference in elevation of density optima (positive or negative). Species with a significant difference indicated by non-overlapping confidence intervals are shown in bold. ^E^ indicates endemic species, ^S^ indicates northern and southern populations have subspecific status.

### Comparing observed and predicted elevational differences

**Figure 4:**
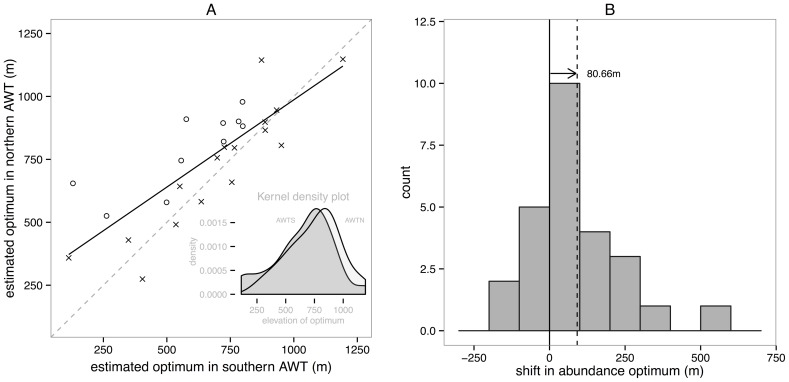
Trends among sensitive species in differences in the elevation of density optima. **A)** Differences in the elevation of density optima between southern and northern AWT bird populations. Data are elevations of density optima estimated for species for which Gaussian response curves were identified as the best fit using AIC in the HOF approach, recalculated with confidence intervals using the approach of Oksanen et al. [21]. The diagonal dashed line shows the line of no shift between subregions, while the solid line is a simple linear model fit to the density optimum data (r^2^ = 0.633, f = 44.22, d.f. = 24, p = <0.001). Species whose southern upper and northern lower 84% confidence intervals do not overlap are indicated with open circles. The inset figure shows kernel density plots of the elevations of species elevation optima in the southern AWT overlayed on those for the northern AWT, illustrating the upward displacement in the central tendency of these values with latitude. **B)** Distribution of differences between the elevation of density optima fitted to Gaussian response-species between the southern and northern AWT regions. The vertical lines separated by an arrow indicate the difference between zero (no shift) and the Wilcoxon test of median difference between southern and northern AWT optima values across the 26 taxa (+80.66 m).

Among the species we identified as likely to be temperature sensitive, positive elevational differences in density optima between the southern and northern AWT drove a consistent trend upslope relative to a line of no difference (Fig. 4A). An overlay of the kernel density plots of optima elevations in the two regions illustrates the distribution of optima across the elevational gradient, and the offset between subregions. Fig. 4B also shows the positive (upslope) bias in the median value of shifts across these 26 species. The results of a series of non-parametric tests for the significance of these shifts are shown in Table 3. A Wilcoxon signed rank test indicated a significant positive median difference in optima elevations for northern populations relative to their southern counterparts of 80.66 m (p-value for H1: the median difference is not equal to zero  = 0.006, n1  =  n2  = 26), which was not significantly different from the bioCLIM estimated Mean Annual Temperature displacement of 75.56 m p-value (for H1: the median difference is not equal to 75.5 m = 0.86). While the observed median difference was not consistent with predictions based on bioCLIM T_max_ of a downward displacement (predicted  = −81.17 m), nor with the greater up-slope shift predicted by bioCLIM T_min_ (predicted  = +403 m), the lower interquartile range of species profile differences between southern AWT and northern AWT includes some down-slope-shifts (observed  = −75.3 m) and is consistent with predictions based on T_max_ (Table 3). AccuCLIM MAT did not provide a better prediction of this median shift across all species (+54.89 m, P = 0.33) and nor did the corresponding accuCLIM T_max_ and T_min_ (Table 3). Root Mean Squared Errors for regressions of observed and expected optima values, however, showed a marginal advantage of accuCLIM (RMSE 143.7) over bioCLIM (RMSE  = 146.2) for MAT, though not for T_max_ and T_min_ (Table 3).

**Table 3:** Results of Wilcoxon nonparametric tests for differences in location of species’ density optima.

Climate surface	Temperature Parameter	Predicted temperature difference (°C)	Predicted altitude difference(m)	Observed altitude difference (m)	Wilcoxon P-value	RMSE
**bioCLIM**	MAT	0.41	75.57	80.89	**0.84**	146.19
	T_max_	−0.38	−81.18	−75.31	**0.94**	255.27
	T_min_	1.77	403.58	261.97	0.05	241.74
**accuCLIM**	MAT	0.35	54.89	80.89	**0.33**	143.70
	T_max_	−1.47	−171.68	−75.31	0.02	340.59
	T_min_	2.10	487.58	261.97	0.03	249.98

Comparisons of the locations of elevational density optima between southern and northern AWT relative to predicted values based on MAT, T_max_ and T_min_ from both bioCLIM and accuCLIM. P-values are the results of Wilcoxon tests of the null hypothesis of no difference between the observed location differences and those predicted based on the corresponding temperature gradient across elevation in each case. Tests that failed to reject H_0_ are shown in bold. RMSE values are those associated with a simple linear model of each species’ observed density optima against the corresponding predicted values.

## Discussion

Of the 80 rainforest bird species examined in detail this study, 47 (59%) exhibited a unimodal (Gaussian or skewed) density response across the temperature gradient, and 26 were amenable to analysis using the Oksanen et al. [22] method for calculating the location and confidence intervals of elevational density optima. Of this subset of tractable species, 19 (73%) exhibited a positive displacement in peak density between southern and northern AWT, driving a significant trend across this sensitive subset of the avifauna that matches expectations based on local elevational temperature gradients. These results suggest that differences in temperature provide a parsimonious explanation for spatial variation in elevational distributions of a substantial proportion of the rainforest bird assemblage in this system. In addition these results show that temperature sensitivities are conserved between populations, such that density profiles in southern and northern AWT spatial subsets responded in a predictable fashion to the effect of latitude on elevational temperature gradients, rather than idiosyncratically. While concentrated in mid-elevations (as is species diversity in the study region [35]), these differences were also found across the elevational gradient, indicating that temperature sensitivity is not limited to the cool-adapted upland species previously considered most vulnerable to climate change [9]. Interestingly, species exhibiting shifts ranged from restricted endemic, insectivorous under-storey passerines (e.g. Fernwren, *Oreoscopus gutturalis*) to widespread, non-passerine canopy frugivores (e.g. Brown Cuckoo-Dove, *Macropygia amboinensis*), and we found no evidence for taxonomic, biogeographic or ecological correlates of either temperature response types or the presence of a positive displacement, further suggesting that unimodal responses may be prevalent across this assemblage, and not restricted to a particular subset of species such as upland endemics.

Narrow thermal tolerances have been recognised as a common feature of the tropical ectotherm biota [6], [43] but there is little empirical data to support temperature as a critical determinant of distributions in tropical endotherms such as birds [44]. The metabolic and water costs of endothermy, however, may expose birds to risks from elevated temperature similar to those predicted for ectotherms [45]. The assumption that species distributions are strongly influenced by climate, and in particular temperature, is critical when modelling those distributions as a function of climate variables, as is common in projections of the future impact of climate change [46], [47]. Despite the widespread application of such models, including to future predictions of rainforest bird distributions in this system [9], [28], historical data with which to test these assumptions are often lacking, and while desirable, empirical studies of temperature sensitivity are costly, and rare [6]. The space-for-time substitution approach taken in this study thus constitutes an important evaluation of the assumption of temperature limitation and niche transferability among sensitive species in a tropical rainforest avifauna. This is in contrast to studies that have found little limitation of distribution by climate in other avifaunas (e.g. [48]), and supports arguments for continued careful use of correlative distribution models in predicting climate change impacts [49].

### Monitoring of range shifts

The data and analytical approach presented here for quantifying elevational density profiles of species addresses important gaps in our understanding of climate-related impacts in this diverse tropical system. First, we have used distance sampling to provide baseline estimates of density corrected for differences in detectability between sites and species. Absolute density provides a more robust measure of species’ abundance responses to environmental gradients by controlling for the effects of extraneous factors such as differences in habitat structure, which may be influential across large environmental gradients. Distance sampling or similar approaches are therefore also expected to be critical for quantifying important changes in population size resulting from range shifts. Second, we have improved on previous efforts to quantify elevational abundance responses [9], [50] or elevational position of bird distributions using basic measures of central tendency [18]. We have shown that elevational optima can be derived for a substantial portion of species using simple Gaussian response curves, and that these can be employed to document modest upslope shifts involving temperature differences of as little as 0.41°C. This would suggest a high utility of this analytical approach in documenting early change in this avifaunal community. Importantly, this magnitude of change is also within the range predicted for the AWT within 20 years under current warming trends [51].

### Other drivers of elevational differences

Variation in magnitude of up-slope shifts between species shown here echoes findings in temporal studies of range-shift [11], [12]. Variation in species characteristics may be an important driver of such differences in temperature response [52]. The sensitivity of species to environmental gradients may vary between species depending on their behaviour or physiology; for example, migration phenology and diel rhythms may influence the actual temperatures experienced by individuals, or alter their capacity for buffering against temperature variation [53]. Depending on their physiologies, species may also be more sensitive to temperature maxima or minima rather than means [44], [54]. While the median displacements we report are consistent with our expectations, we also document a range of responses which includes some greater upslope shifts, and even some down-slope shifts. We show evidence among some species for downslope shifts consistent with predictions based on maximum temperatures as a limiting factor, paradoxically in this case as the local temperature lapse is reversed for T_max_ (likely lowered in the north by the closer proximity of our montane study sites to coastal influences). Elevational gradients are also complex, and include interactions between temperature, habitat, rainfall and seasonality [55], so species’ responses may differ in cases of sensitivity to gradients other than temperature. Rainfall seasonality in particular varies across the elevational gradient in this study, and may also play a role in determining species distributions. For example, extreme rainfall events at high elevations have been shown to drive some species down-slope [56], and sensitivity may vary between species.

Down-slope shifts in species distributions have also been documented elsewhere as a result of climate change [11], [12]. Habitat modification, competitive interactions and the influence of climate variables other than mean temperature have all been identified as possible drivers of such unexpected reversals of the overall up-slope trend in shifts [50]. We suggest that habitat modification is unlikely to be important in the system studied here, as there is minimal impact over much of the elevational gradient [58], and no systematic variation between the two regions compared. The interactions with competitors which may also influence species distributions [59] are also unlikely to be responsible in this case, as there is little assemblage change over the sub-regional scale examined here [60]. As in the case of variation between species’ up-slope shifts, influence of environmental factors besides broad scale estimates of mean annual temperature may however be important in driving down-slope shifts. Downslope shifts could result when the processes determining upper and lower range boundaries differ [61], for example through trade-offs between life-history traits and metabolic costs [62].

Despite these potentially confounding influences, however, here we show an upward trend in the latitudinal displacement of elevational density optima that is highly consistent with expectations derived from a simple model using only mean annual temperature. This parsimonious model of species elevational density response has the additional advantage of being more readily extrapolated to future climate scenarios, for which projections in the study area are uncertain [57]. Finally, it has been suggested that the use of measures that better capture the conditions experienced by individual organisms, such as under-canopy temperature data, may help to clarify the role of temperature in driving species distributions [63]. Interestingly we show that while under-canopy temperatures from accuCLIM here provided a slight improvement in RMSE for regressions of observed and predicted optima, a simple model using only Mean Annual Temperature from broad-scale climate surfaces performed well as a predictor of species’ median responses.

### Limitations of the approach

An important limitation of our analysis is the reduced utility of the HOF approach [30] for characterising the responses of species whose optima approach the limits of the environmental gradient. This reduces the scope of the analysis by limiting the number of species amenable to testing, excluding some taxa at both extremes of the thermal gradient in the study region, as temperature responses in these species tended to be identified by the HOF approach as having monotonic or plateau responses. Importantly, however, rather than concluding that these species are responding altogether differently to temperature (an assumption for which there is little theoretical basis), these responses may best be described as some fraction of a unimodal curve whose optimum is truncated by the gradient limits. From a monitoring perspective, these species may be equally sensitive to changes in temperature, but detecting their response may instead require an ensemble of approaches including comparison of absolute density through time (e.g. differences between intercepts of monotonic responses) or changes in the location of nick-points (in the case of plateau responses). Additionally, even among unimodal responses, problems may be encountered at domain boundaries when fitting the Gaussian curve with confidence intervals using the approach of Oksanen et al. [22]. In order to have sufficient data to accurately describe both the increase and decrease phases, we suggest that higher sampling intensity at distribution limits should be a priority in optimal programs for the monitoring of climate-induced range shifts in montane species.

Skewed responses were also relatively common in the results presented here. A Gaussian curve is often assumed to be the underlying distribution in species’ responses to environmental gradients, but there are also physiological and ecological reasons to expect skewed distributions [40]. The parameterisation of such non-symmetrical responses is a recurring issue in community ecology [21], where inter-specific interactions and metabolic constraints may drive asymmetry in gradient responses [64]. While it is beyond the scope of the analyses presented here, a systematic examination of the profiles of species with and without the presence of potential competitors may allow the assessment of the extent to which competition (for example) may contribute to skewed responses in this system. The fact that many of the optima identified by skewed distributions deviated little from the corresponding symmetrical Gaussian distribution for that species (see e.g. Fig. S3.30: Grey Fantail (*Rhipidura albiscapa),* Fig. S3.12: Bowers Shrike-thrush (*Colluricincla bowerii)*, and Fig. S3.14: Bridled Honeyeater (*Lichenostomus frenatus*), suggests, however, that this analytical limitation does not alter the overall conclusions of our analysis. More complex approaches such as those using Generalised Additive Models (GAM) have also been suggested where multiple drivers are suspected to be important (e.g. [65], but these models may be more difficult to transfer in space and time, limiting their utility in the detection of distribution changes [66]. Finally, there are some cases in which density may not be a good indicator of environmental optima, such as among sink populations [67], [68]. While data are available on physiological limits among reptiles and amphibians in the Wet Tropics, few direct measures of environmental tolerances of tropical birds have been attempted [69], so limiting our analysis to the correlations we present above. We suggest, however, that in the absence of alternatives, density optima provide a useful, data-efficient and cost-effective approximation. A mechanistic corroboration of this result would be a useful future investigation, but our results demonstrate a parsimonious approach to detection and monitoring of change without the need for more time-consuming or invasive procedures, critical factors in the timely monitoring of vulnerable and restricted species.

### Conclusions and further work

Despite some complexities, we document a coherent latitudinal signature of positive difference in elevation of density optima among rainforest birds in this system, which is consistent with expectations from a simple hypothesis based on mean annual temperature derived from broad-scale climate surfaces. While our sample includes many species from the diverse mid-slopes of the study area, differences are also found among species in megatherm lowland environments. This tendency for lowland species to respond similarly to increases in temperature to their upland counterparts may reflect a general tendency toward narrow thermal tolerance in tropical species [43], and has important repercussions for lowland biodiversity in a changing climate [6]. In some situations [5], predicted upslope shifts of lowland species driven by thermal tolerances could result in a process of lowland biotic attrition in the montane tropics [70]. The data we present here suggest that the assumption of temperature dependency underlying this prediction may be reasonable for a substantial proportion of both the lowland and upland avifauna in the Wet Tropics.

Globally, montane rainforest birds are at high risk from the warming associated with climate change [14], [71]. Unfortunately, in most cases elevational range information for tropical montane birds is limited to coarse estimates based on presence records. Such data may be useful in larger scale studies [72], but a lack of fine-scale information may have contributed to a failure to detect recent impacts despite documented climate change elsewhere [73]. The early detection of shifts necessary for effective conservation management in the face of global warming requires information over short spatial or temporal scales [18]. We demonstrate here an approach to collecting such data to derive region-wide estimates of optima for a diverse tropical community. Our results also demonstrate that these data can be used to predict and detect elevational range-shifts at fine spatial and temporal scale, and suggest a method for collection and analysis of further baseline data in this system to build on existing information. As evidence validating the assumption of a temperature limitation on some species distributions, results such as these also lend support to predictions from correlative distribution modeling [9] that climate change will have profound impacts on the biodiversity of the montane rainforest bird fauna in northeastern Australia and elsewhere. We therefore encourage development of other similar data sets to address the deficit of global change studies in vulnerable montane avifaunas of the tropics.

## Supporting Information

Figure S1 **Scatter plots of the relationship between temperature parameters and elevation in the study area for accuCLIM.** Results are shown for MAT, T_max_ and T_min_ derived from microclimate measured across the range of elevations present in the southern AWT (filled circles) and northern AWT (unfilled circles). Solid lines are simple linear models of the effect of elevation on temperature for each parameter, with the trend for southern sites shown by a solid line, that for northern sites with a dashed line. See main text for an explanation and reference for the methods used to derive these data.
(PDF)

Figure S2 **Results of the Huisman-Olff-Frescoe (HOF) hierarchical model fitting process.** Models are shown for rainforest bird density responses across the temperature gradient in the study region. Models tested were flat (light blue), plateau (green), monotonic (dark blue), unimodal (Gaussian) (red) and skewed (black). AIC values (upper right of each plot) were used to select the most appropriate model in each case (lines shown in bold in each case).
(PDF)

Figure S3 **Example fitted Gaussian curves.** Gaussian curves (dashed lines) are shown fitted to the elevational density profiles for the remaining species examined for elevational difference in their estimated density optima between southern AWT (filled circles) and northern AWT (unfilled circles). Data are the estimated densities calculated with Distance analysis at each sampling point across the elevational gradient. Arrows and their labels indicate the direction and magnitude of the elevational shift. See Table 2 for model parameters and tests of significance relating to these observed shifts.
(PDF)

Table S1 **AIC scores for competing models in HOF** [**30**] **analysis.** Shown are competing models in a hierarchical Huissman-Olff-Frescoe [30] model selection analysis for elevational density responses across the 88 Australian Wet Tropics rainforest bird species (those with sufficient sampling in this study). Models were selected using the approach implemented in the R package “BiodiversityR” (Kindt 2011) (see methods for details).
(PDF)
