# Supplementary material for: Current Analogues of Future Climate Indicate the Likely Response of a Sensitive Montane Tropical Avifauna to a Warming World
Source: PLoS One. 2013 Jul 31;8(7):e69393. doi: 10.1371/journal.pone.0069393 (PMC3729957; doi:10.1371/journal.pone.0069393)
Supplement: Figure S1 — Scatter plots of the relationship between temperature parameters and elevation in the study area for accuCLIM. Results are shown for MAT, Tmax and Tmin derived from microclimate measured across the range of elevations present in the southern AWT (filled circles) and northern AWT (unfilled circles). Solid lines are simple linear models of the effect of elevation on temperature for each parameter, with the trend for southern sites shown by a solid line, that for northern sites with a dashed line. See main text for an explanation and reference for the methods used to derive these data. (PDF) [file pone.0069393.s001.pdf]

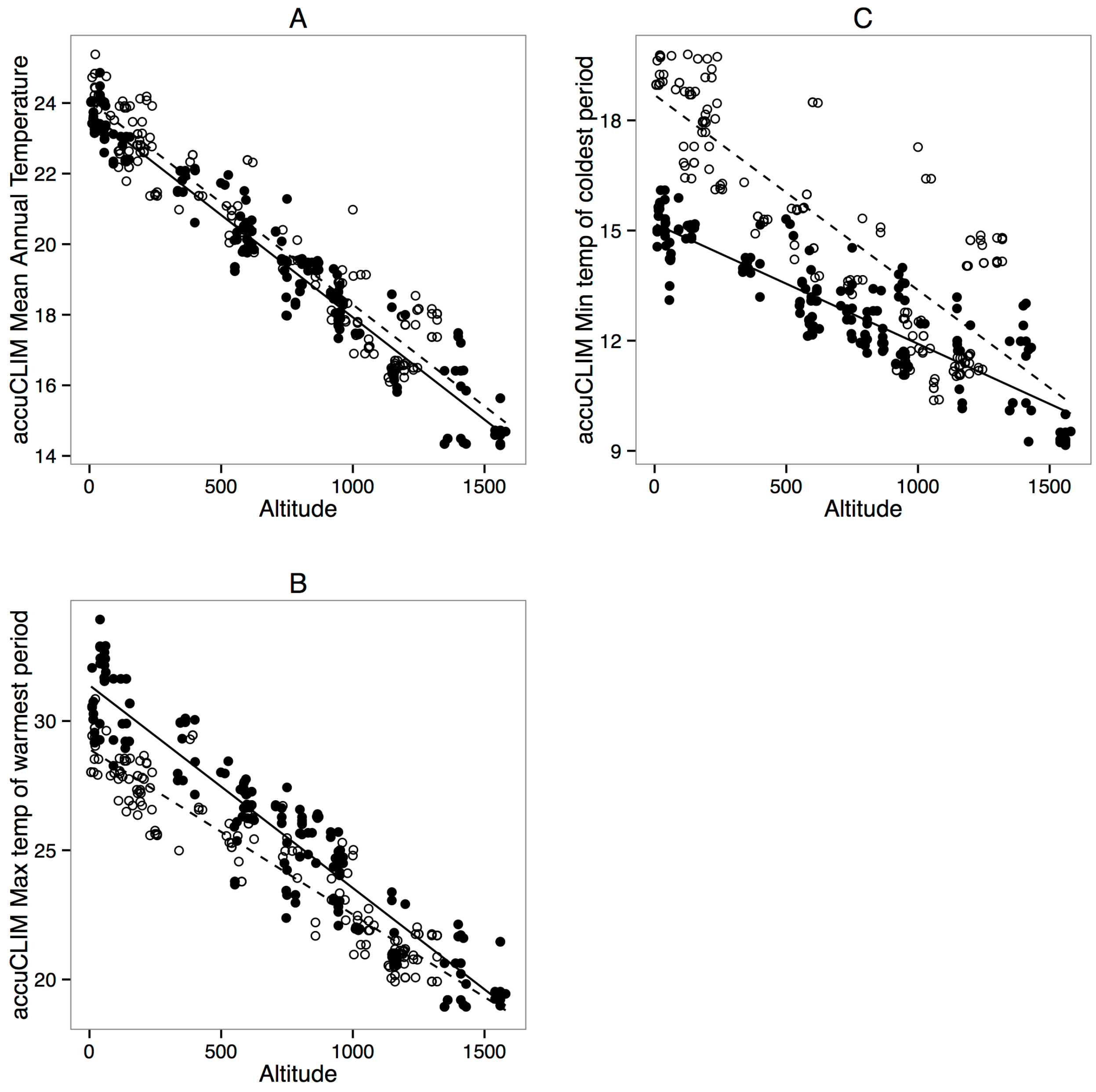


**Figure S1. Scatter plots of the relationship between temperature parameters and elevation in the study area for accuCLIM**. Results are shown for MAT, $T_{max}$ and $T_{min}$ derived from microclimate measured across the range of elevations present in the southern AWT (filled circles) and northern AWT (unfilled circles). Solid lines are simple linear models of the effect of elevation on temperature for each parameter, with the trend for southern sites shown by a solid line, that for northern sites with a dashed line. See main text for an explanation and reference for the methods used to derive these data.
