# Supplementary material for: Current Analogues of Future Climate Indicate the Likely Response of a Sensitive Montane Tropical Avifauna to a Warming World
Source: PLoS One. 2013 Jul 31;8(7):e69393. doi: 10.1371/journal.pone.0069393 (PMC3729957; doi:10.1371/journal.pone.0069393)
Supplement: Figure S2 — Results of the Huisman-Olff-Frescoe (HOF) hierarchical model fitting process. Models are shown for rainforest bird density responses across the temperature gradient in the study region. Models tested were flat (light blue), plateau (green), monotonic (dark blue), unimodal (Gaussian) (red) and skewed (black). AIC values (upper right of each plot) were used to select the most appropriate model in each case (lines shown in bold in each case). (PDF) [file pone.0069393.s002.pdf]

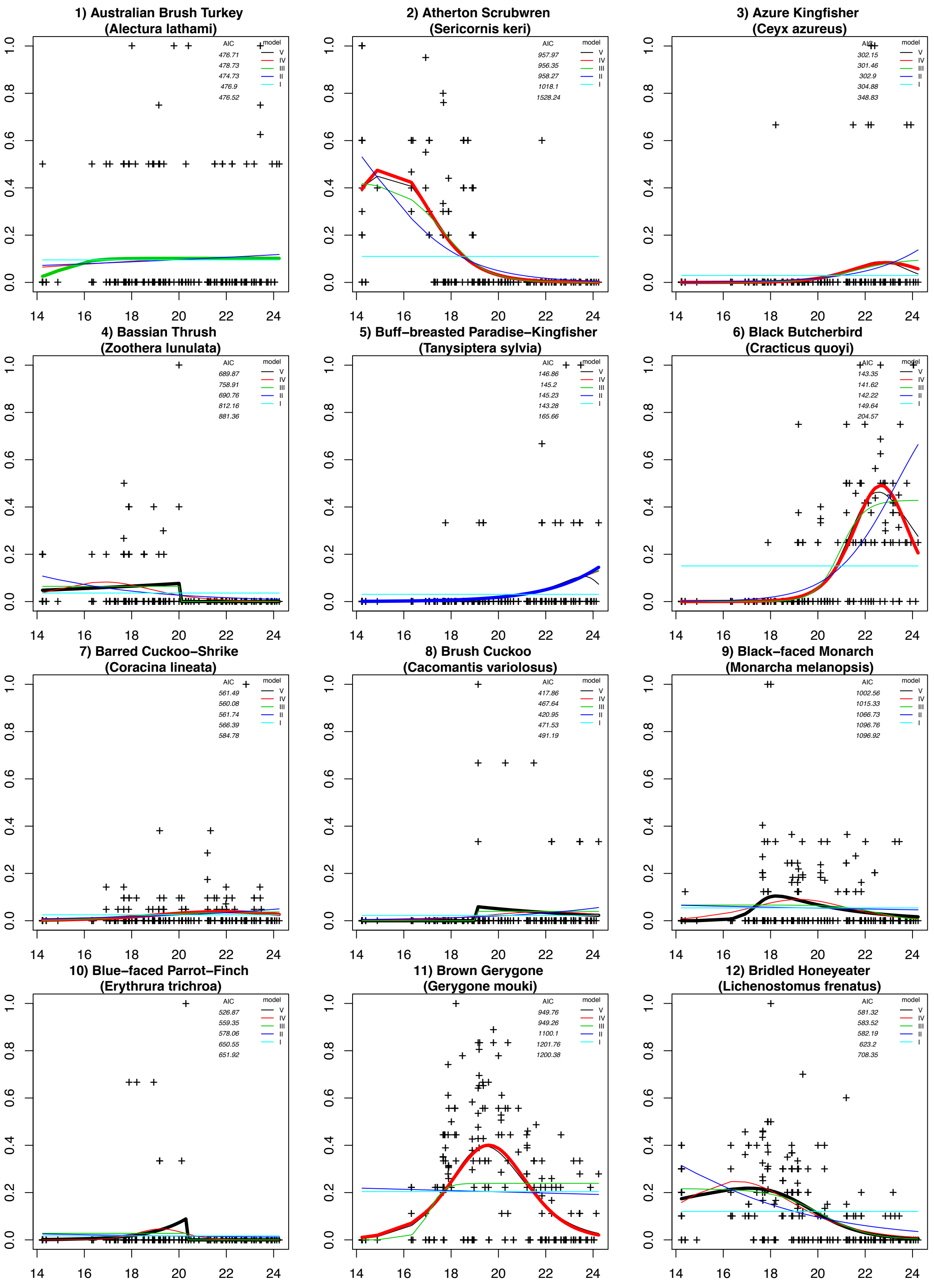

Figure S2. Results of the Huisman-Olff-Frescoe (HOF) hierarchical model fitting process. Models are shown for rainforest bird density responses across the temperature gradient in the study region. Models tested were flat (light blue), plateau (green), monotonic (dark blue), unimodal (Gaussian) (red) and skewed (black). AIC values (upper right of each plot) were used to select the most appropriate model in each case (plotted lines shown in bold).

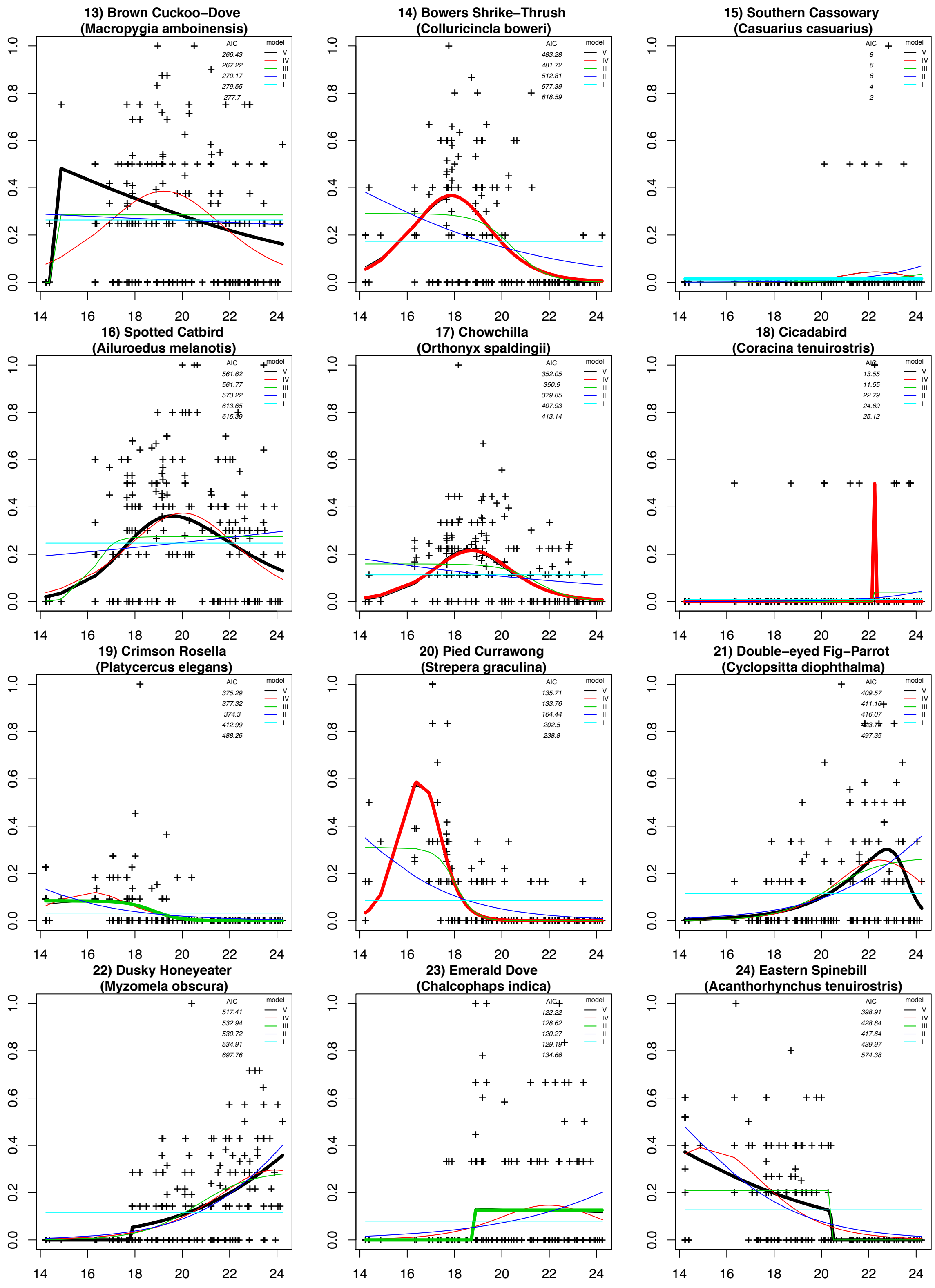

Figure S2 (CONTINUED). Results of the Huisman-Olff-Frescoe (HOF) hierarchical model fitting process. Models are shown for rainforest bird density responses across the temperature gradient in the study region. Models tested were flat (light blue), plateau (green), monotonic (dark blue), unimodal (Gaussian) (red) and skewed (black). AIC values (upper right of each plot) were used to select the most appropriate model in each case (plotted lines shown in bold).

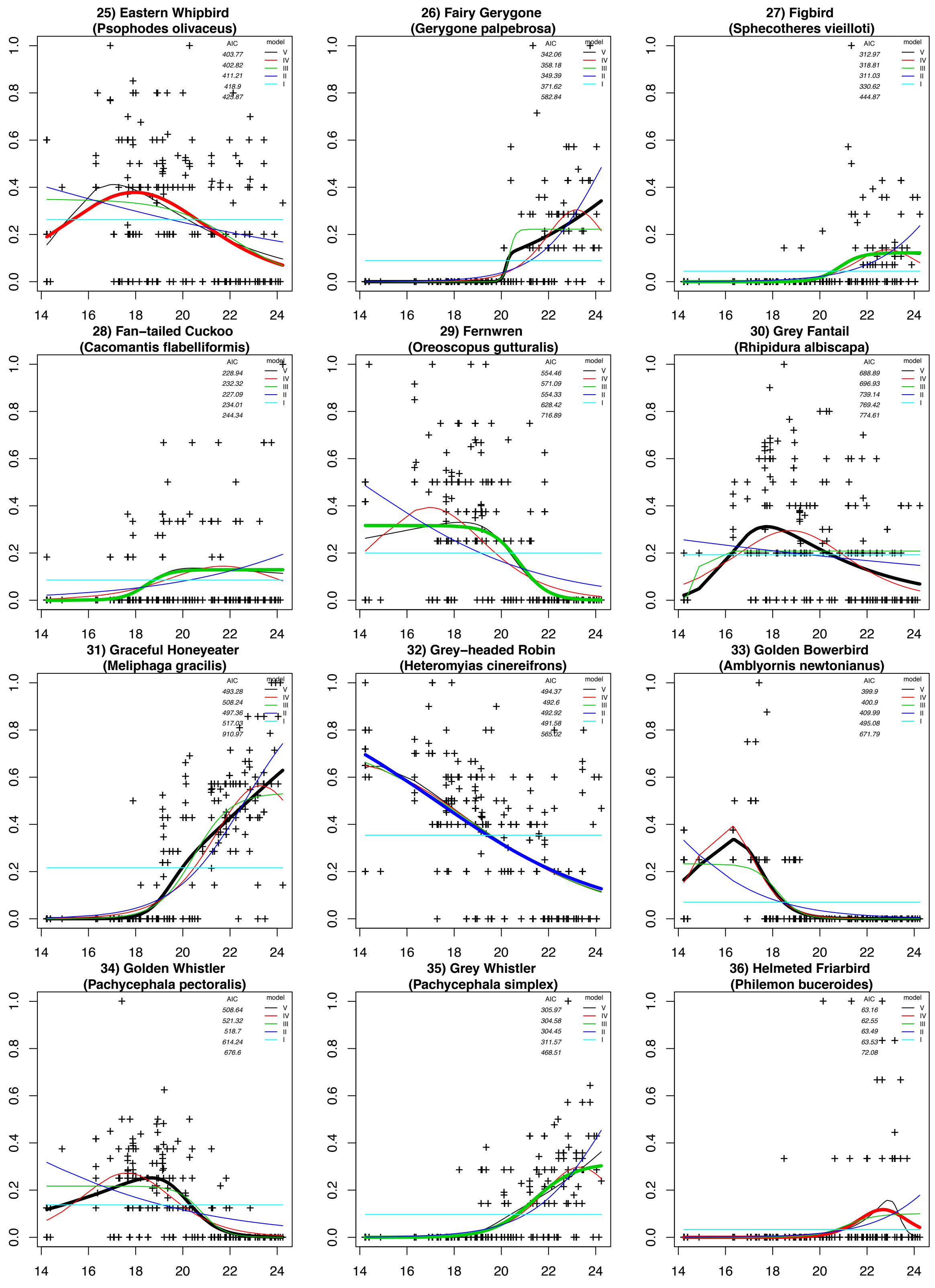

Figure S2 (CONTINUED). Results of the Huisman-Olff-Frescoe (HOF) hierarchical model fitting process. Models are shown for rainforest bird density responses across the temperature gradient in the study region. Models tested were flat (light blue), plateau (green), monotonic (dark blue), unimodal (Gaussian) (red) and skewed (black). AIC values (upper right of each plot) were used to select the most appropriate model in each case (plotted lines shown in bold).

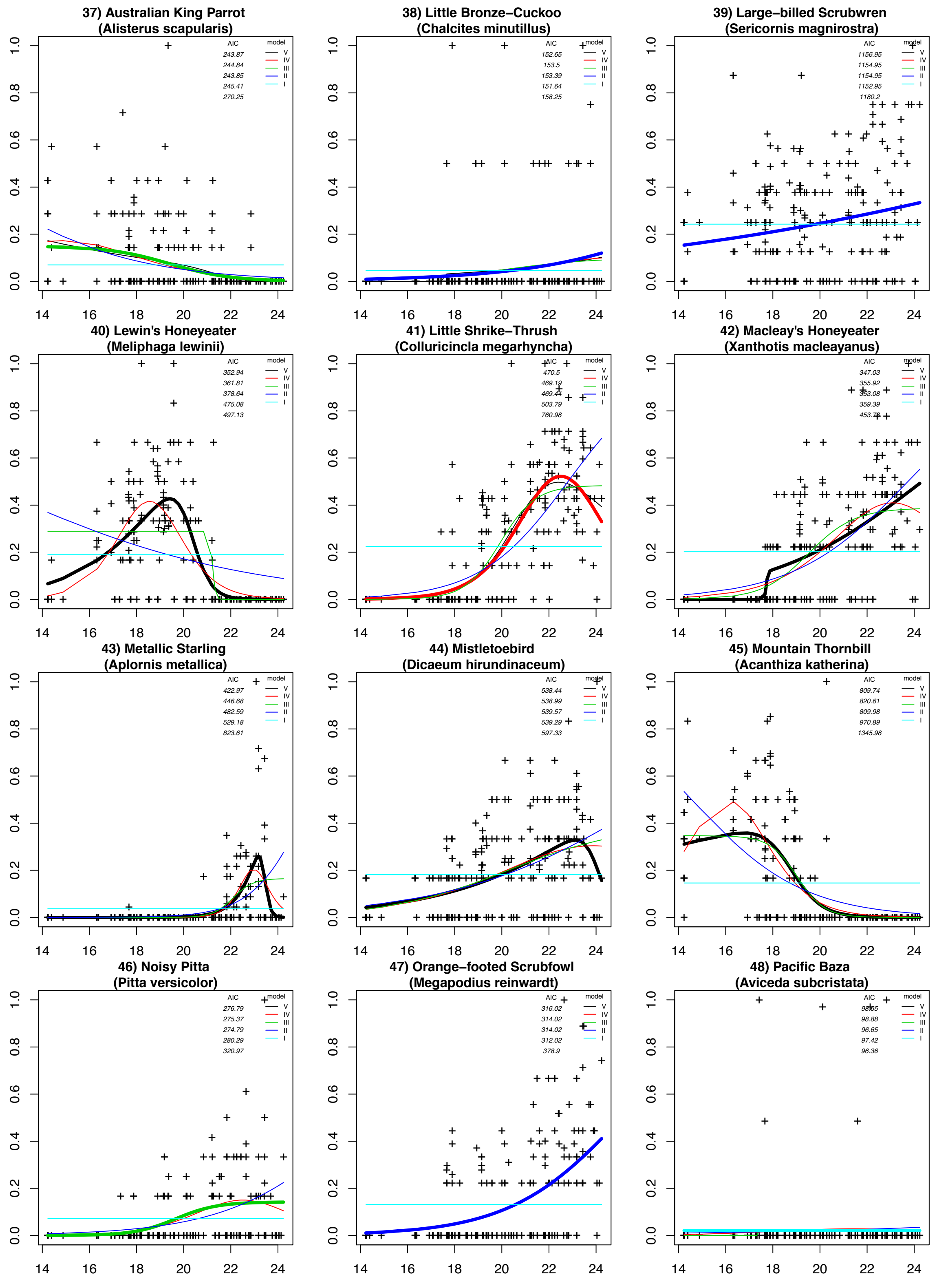

Figure S2 (CONTINUED). Results of the Huisman-Olff-Frescoe (HOF) hierarchical model fitting process. Models are shown for rainforest bird density responses across the temperature gradient in the study region. Models tested were flat (light blue), plateau (green), monotonic (dark blue), unimodal (Gaussian) (red) and skewed (black). AIC values (upper right of each plot) were used to select the most appropriate model in each case (plotted lines shown in bold).

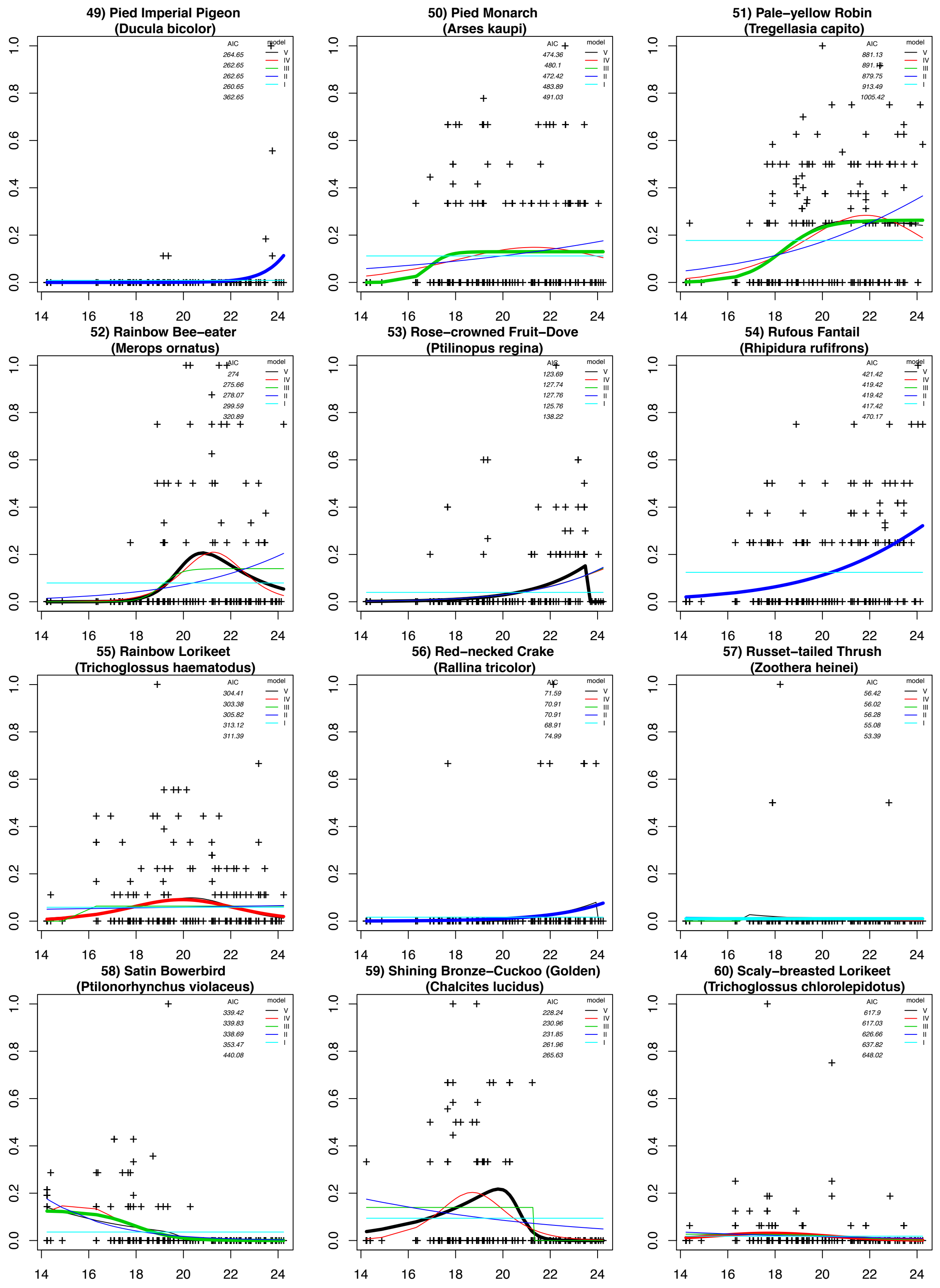

Figure S2 (CONTINUED). Results of the Huisman-Olff-Frescoe (HOF) hierarchical model fitting process. Models are shown for rainforest bird density responses across the temperature gradient in the study region. Models tested were flat (light blue), plateau (green), monotonic (dark blue), unimodal (Gaussian) (red) and skewed (black). AIC values (upper right of each plot) were used to select the most appropriate model in each case (plotted lines shown in bold).

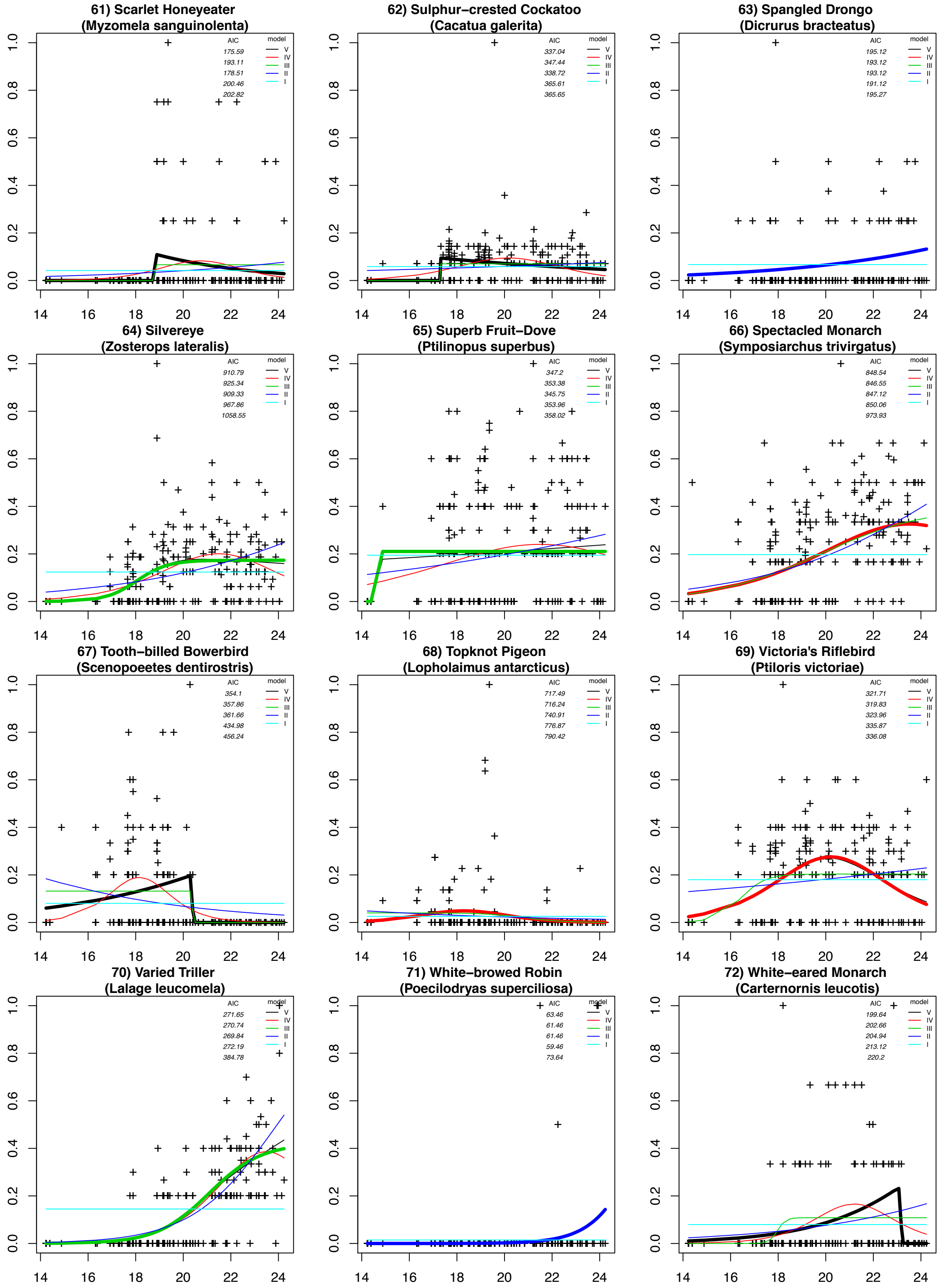

Figure S2 (CONTINUED). Results of the Huisman-Olff-Frescoe (HOF) hierarchical model fitting process. Models are shown for rainforest bird density responses across the temperature gradient in the study region. Models tested were flat (light blue), plateau (green), monotonic (dark blue), unimodal (Gaussian) (red) and skewed (black). AIC values (upper right of each plot) were used to select the most appropriate model in each case (plotted lines shown in bold).

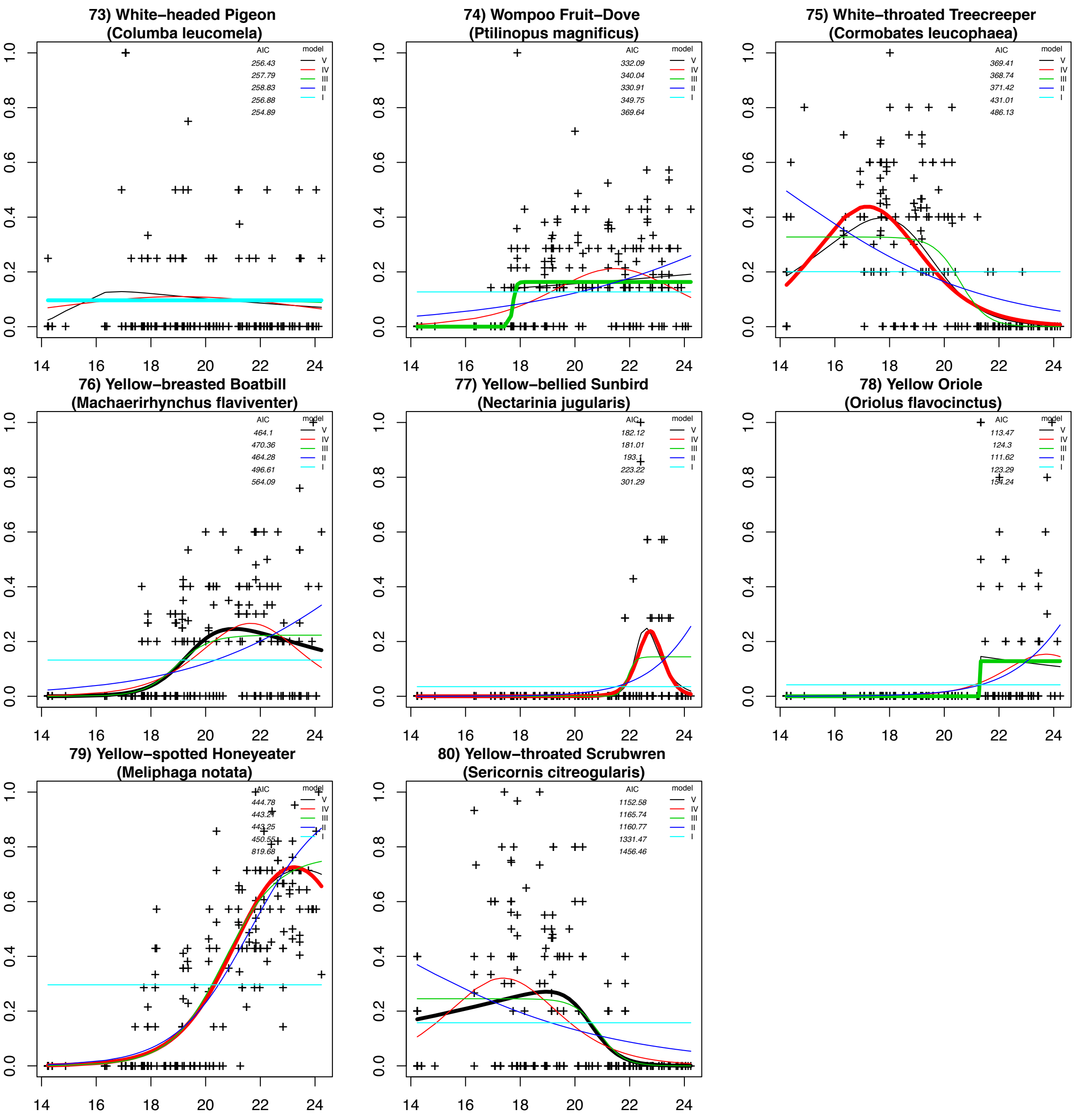

Figure S2 (CONTINUED). Results of the Huisman-Olff-Frescoe (HOF) hierarchical model fitting process. Models are shown for rainforest bird density responses across the temperature gradient in the study region. Models tested were flat (light blue), plateau (green), monotonic (dark blue), unimodal (Gaussian) (red) and skewed (black). AIC values (upper right of each plot) were used to select the most appropriate model in each case (plotted lines shown in bold).
