# Supplementary material for: Current Analogues of Future Climate Indicate the Likely Response of a Sensitive Montane Tropical Avifauna to a Warming World
Source: PLoS One. 2013 Jul 31;8(7):e69393. doi: 10.1371/journal.pone.0069393 (PMC3729957; doi:10.1371/journal.pone.0069393)
Supplement: Figure S3 — Example fitted Gaussian curves. Gaussian curves (dashed lines) are shown fitted to the elevational density profiles for the remaining species examined for elevational difference in their estimated density optima between southern AWT (filled circles) and northern AWT (unfilled circles). Data are the estimated densities calculated with Distance analysis at each sampling point across the elevational gradient. Arrows and their labels indicate the direction and magnitude of the elevational shift. See Table 2 for model parameters and tests of significance relating to these observed shifts. (PDF) [file pone.0069393.s003.pdf]

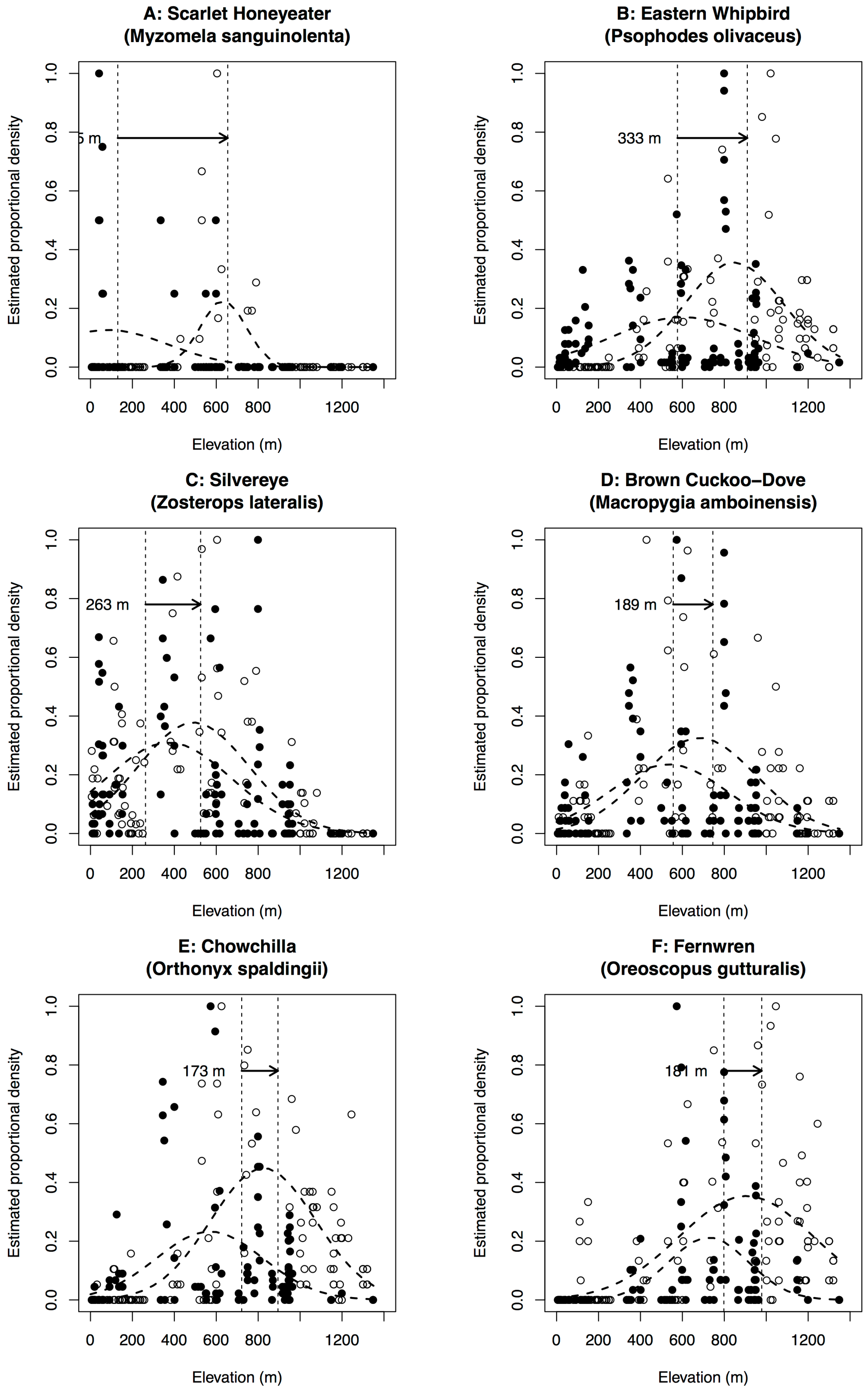

Figure S3. Example fitted Gaussian curves. Gaussian curves (dashed lines) are shown fitted to the elevational density profiles for the remaining species examined for elevational difference in their estimated density optima between southern AWT (filled circles) and northern AWT (unfilled circles). Data are the estimated densities calculated with Distance analysis at each sampling point across the elevational gradient. Arrows and their labels indicate the direction and magnitude of the elevational shift. See Table 2 for model parameters and tests of significance relating to these observed shifts.

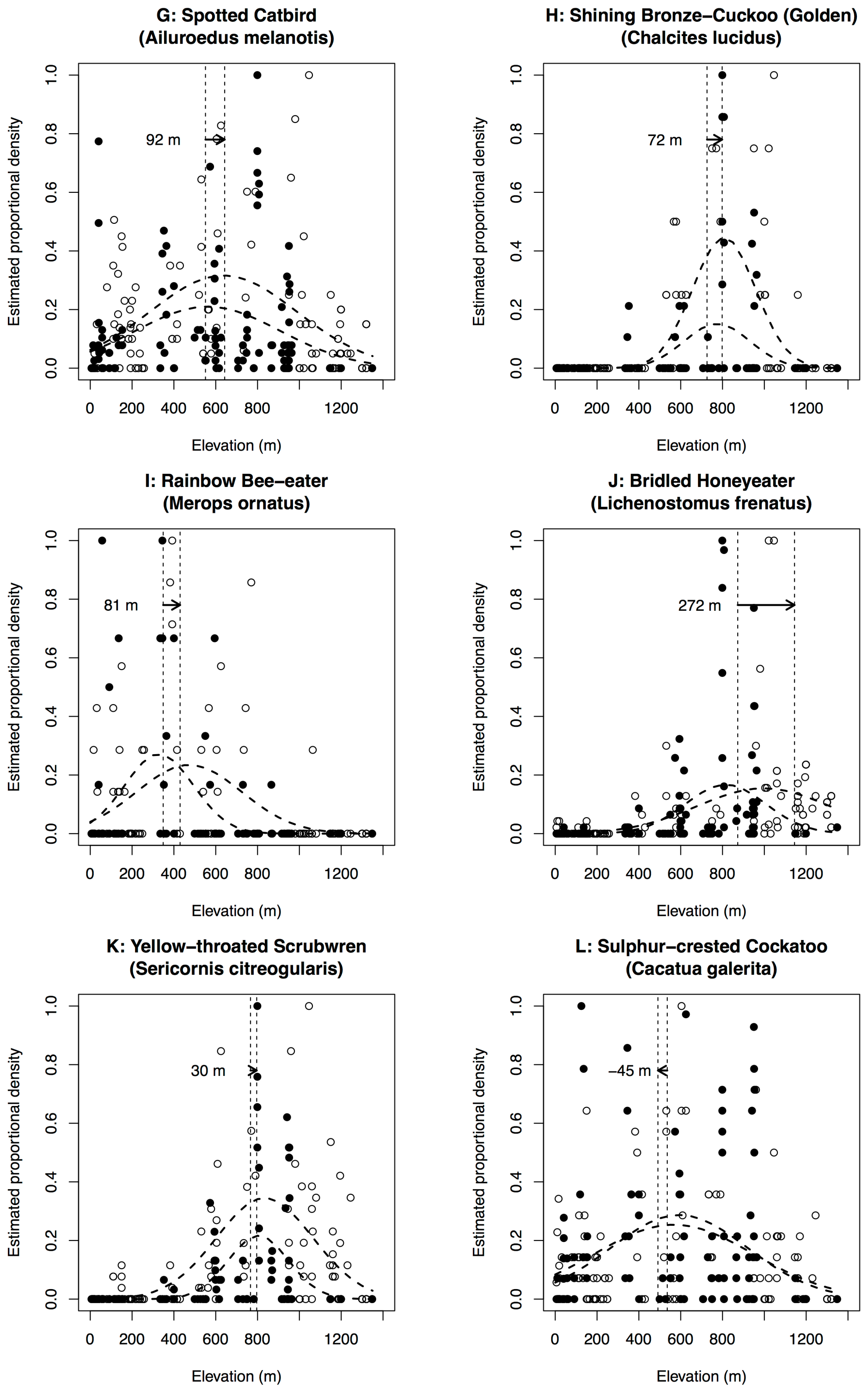

Figure S3 (CONTINUED). Example fitted Gaussian curves. Gaussian curves (dashed lines) are shown fitted to the elevational density profiles for the remaining species examined for elevational difference in their estimated density optima between southern AWT (filled circles) and northern AWT (unfilled circles). Data are the estimated densities calculated with Distance analysis at each sampling point across the elevational gradient. Arrows and their labels indicate the direction and magnitude of the elevational shift. See Table 2 for model parameters and tests of significance relating to these observed shifts.

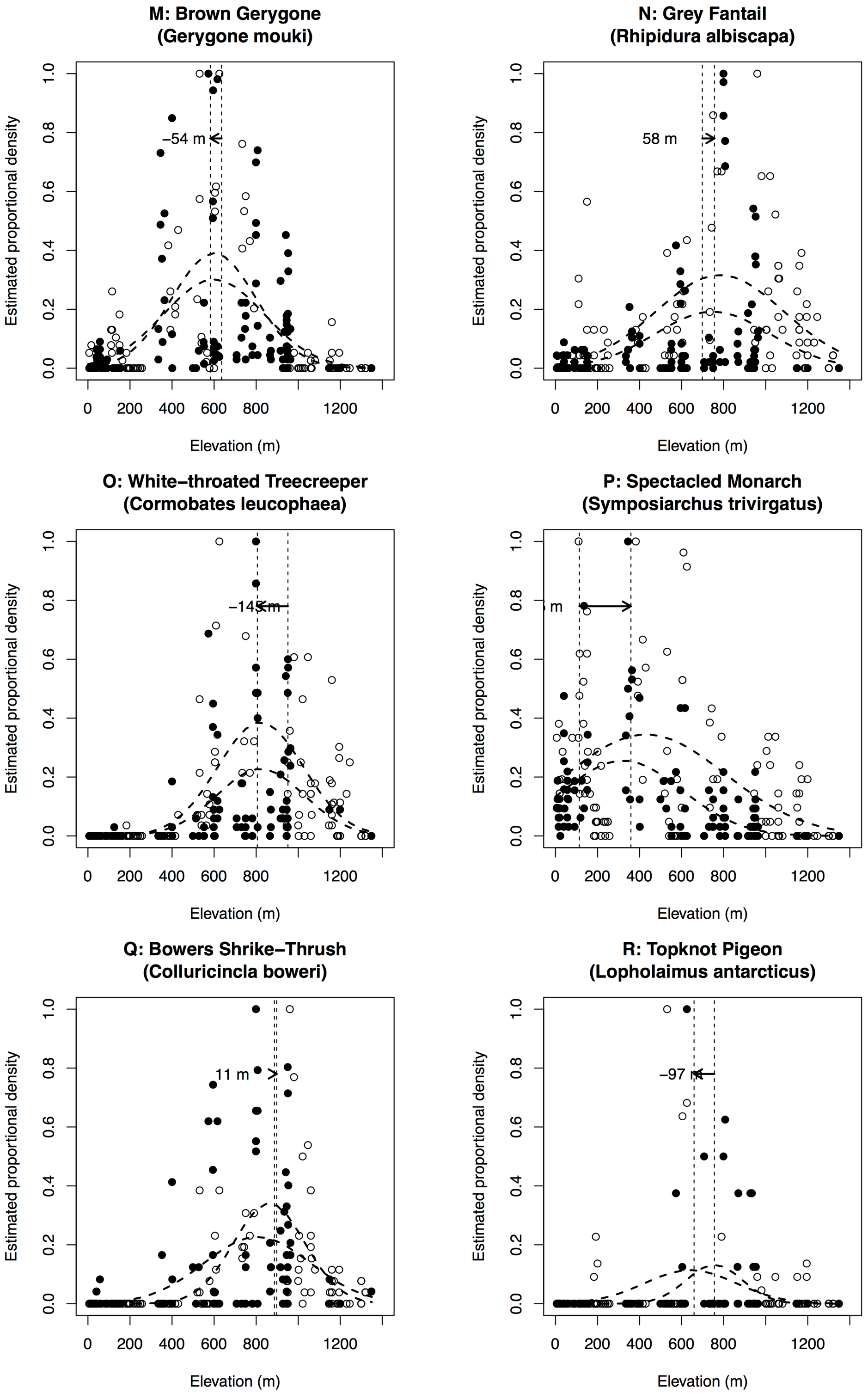

Figure S3 (CONTINUED). Example fitted Gaussian curves. Gaussian curves (dashed lines) are shown fitted to the elevational density profiles for the remaining species examined for elevational difference in their estimated density optima between southern AWT (filled circles) and northern AWT (unfilled circles). Data are the estimated densities calculated with Distance analysis at each sampling point across the elevational gradient. Arrows and their labels indicate the direction and magnitude of the elevational shift. See Table 2 for model parameters and tests of significance relating to these observed shifts.

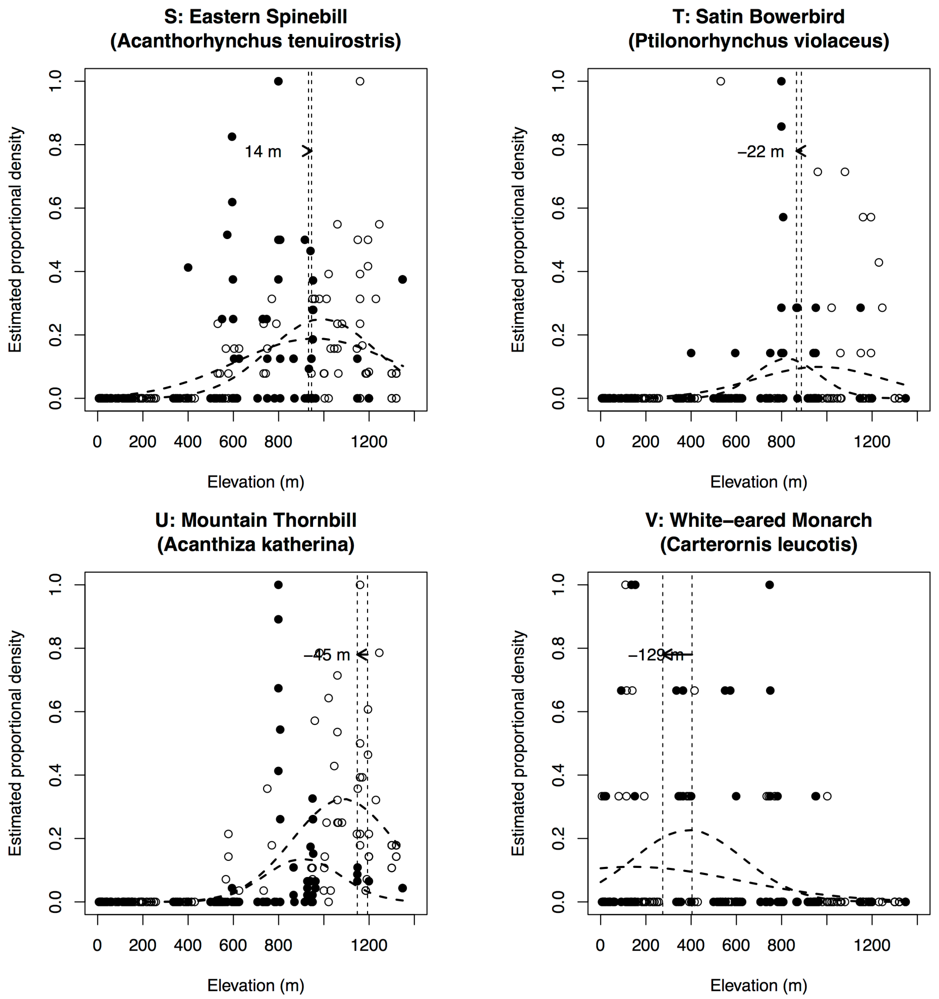

Figure S3 (CONTINUED). Example fitted Gaussian curves. Gaussian curves (dashed lines) are shown fitted to the elevational density profiles for the remaining species examined for elevational difference in their estimated density optima between southern AWT (filled circles) and northern AWT (unfilled circles). Data are the estimated densities calculated with Distance analysis at each sampling point across the elevational gradient. Arrows and their labels indicate the direction and magnitude of the elevational shift. See Table 2 for model parameters and tests of significance relating to these observed shifts.
