# Supplementary material for: Current Analogues of Future Climate Indicate the Likely Response of a Sensitive Montane Tropical Avifauna to a Warming World
Source: PLoS One. 2013 Jul 31;8(7):e69393. doi: 10.1371/journal.pone.0069393 (PMC3729957; doi:10.1371/journal.pone.0069393)
Supplement: Table S1 — AIC scores for competing models in HOF [30] analysis. Shown are competing models in a hierarchical Huissman-Olff-Frescoe [30] model selection analysis for elevational density responses across the 88 Australian Wet Tropics rainforest bird species (those with sufficient sampling in this study). Models were selected using the approach implemented in the R package “BiodiversityR” (Kindt 2011) (see methods for details). (PDF) [file pone.0069393.s004.pdf]

Supplementary material

Table S1. AIC scores for competing models in HOF [30] analysis.

AIC scores

	Species	Model 1 (flat)	Model 2 (monotonic)	Model 3 (plateau)	Model 4 (Gaussian)	Model 5 (skewed)	Top model	Top model ignoring skewness
1	Australian Brush Turkey *(Alectura lathami)*	505.52	504.16	501.54	499.94	501.31	4	4
2	Atherton Scrubwren *(Sericornis keri)*	1677.05	1081.35	1036.95	1037.38	1036.99	3	3
3	Azure Kingfisher *(Ceyx azureus)*	356.66	298.21	297.53	295.10	264.85	5	4
4	Bassian Thrush *(Zoothera lunulata)*	900.11	826.98	750.22	774.48	752.20	3	3
5	Buff-breasted Paradise-Kingfisher *(Tanysiptera sylvia)*	174.61	148.45	150.44	150.44	150.23	2	2
6	Black Butcherbird *(Cracticus quoyi)*	211.72	141.46	140.01	143.46	141.10	3	3
7	Barred Cuckoo-Shrike *(Coracina lineata)*	514.55	499.88	457.73	465.71	459.44	3	3
8	Brush Cuckoo *(Cacomantis variolosus)*	503.08	469.72	417.02	456.97	414.00	5	3
9	Black-faced Monarch *(Monarcha melanopsis)*	1150.70	1150.61	1045.55	1013.66	989.56	5	4
10	Blue-faced Parrot-Finch *(Erythrura trichroa)*	666.00	667.27	590.39	538.15	453.23	5	4
11	Brown Gerygone *(Gerygone mouki)*	1299.02	1289.28	1076.49	884.67	853.18	5	4
12	Bridled Honeyeater *(Lichenostomus frenatus)*	806.58	704.32	669.44	668.08	666.80	5	4
13	Brown Cuckoo-Dove *(Macropygia amboinensis)*	319.75	321.75	310.66	302.21	303.75	4	4
14	Bowers Shrike-Thrush *(Colluricincla boweri)*	698.04	655.03	563.65	521.44	521.09	5	4
15	Southern Cassowary *(Casuarius casuarius)*	2.00	4.00	6.00	6.00	8.00	1	1
16	Spotted Catbird *(Ailuroedus melanotis)*	657.96	652.40	601.72	571.55	570.94	5	4
17	Chowchilla *(Orthonyx spaldingii)*	439.86	434.83	391.83	361.39	363.11	4	4
18	Cicadabird *(Coracina tenuirostris)*	25.49	23.02	22.36	20.30	21.90	4	4
19	Crimson Rosella *(Platycercus elegans)*	551.10	446.05	428.59	430.84	430.54	3	3
20	Pied Currawong *(Strepera graculina)*	267.89	216.26	199.66	195.77	192.63	5	4
21	Double-eyed Fig-Parrot *(Cyclopsitta diophthalma)*	504.61	397.44	399.38	399.42	398.51	2	2
22	Dusky Honeyeater *(Myzomela obscura)*	738.49	539.13	510.49	533.96	508.68	5	3
23	Emerald Dove *(Chalcophaps indica)*	138.30	123.80	115.09	125.24	117.08	3	3

AIC scores

	Species	Model 1 (flat)	Model 2 (monotonic)	Model 3 (plateau)	Model 4 (Gaussian)	Model 5 (skewed)	Top model	Top model ignoring skewness
24	Eastern Spinebill *(Acanthorhynchus tenuirostris)*	659.35	511.10	483.86	499.24	469.55	5	3
25	Eastern Whipbird *(Psophodes olivaceus)*	470.90	465.98	444.92	439.77	441.53	4	4
26	Fairy Gerygone *(Gerygone palpebrosa)*	587.91	346.36	330.40	344.26	317.22	5	3
27	Figbird *(Sphecotheres vieilloti)*	452.02	322.40	298.55	295.75	296.57	4	4
28	Fan-tailed Cuckoo *(Cacomantis flabelliformis)*	259.19	245.69	235.38	238.68	236.71	3	3
29	Fernwren *(Oreoscopus gutturalis)*	802.13	707.56	657.94	668.73	658.57	3	3
30	Grey Fantail *(Rhipidura albiscapa)*	874.79	868.09	828.27	761.19	751.49	5	4
31	Graceful Honeyeater *(Meliphaga gracilis)*	974.78	521.60	478.00	491.57	478.24	3	3
32	Grey-headed Robin *(Heteromyias cinereifrons)*	618.40	547.63	546.48	549.63	547.94	3	3
33	Golden Bowerbird *(Amblyornis newtonianus)*	755.69	495.05	442.02	441.33	441.92	4	4
34	Golden Whistler *(Pachycephala pectoralis)*	759.78	697.36	588.17	562.15	558.70	5	4
35	Grey Whistler *(Pachycephala simplex)*	476.49	286.29	281.08	283.32	281.35	3	3
36	Helmeted Friarbird *(Philemon buceroides)*	73.58	58.20	60.20	60.20	62.20	2	2
37	Australian King Parrot *(Alisterus scapularis)*	294.54	275.78	264.80	270.55	266.11	3	3
38	Little Bronze-Cuckoo *(Chalcites minutillus)*	167.20	158.57	159.09	158.99	160.96	2	2
39	Large-billed Scrubwren *(Sericornis magnirostra)*	1310.62	1268.48	1269.50	1269.56	1271.45	2	2
40	Lewin's Honeyeater *(Meliphaga lewinii)*	565.89	548.39	422.88	361.31	347.95	5	4
41	Little Shrike-Thrush *(Colluricincla megarhyncha)*	816.64	482.92	433.64	447.11	435.34	3	3
42	Macleay's Honeyeater *(Xanthotis macleayanus)*	481.14	357.87	335.08	346.62	332.91	5	3
43	Metallic Starling *(Aplornis metallica)*	851.67	435.83	437.83	437.83	439.83	2	2
44	Mistletoebird *(Dicaeum hirundinaceum)*	626.29	551.96	548.11	549.22	550.07	3	3
45	Mountain Thornbill *(Acanthiza katherina)*	1774.69	1157.98	894.41	861.59	853.01	5	4
46	Noisy Pitta *(Pitta versicolor)*	315.62	258.20	248.20	255.77	250.00	3	3
47	Orange-footed Scrubfowl *(Megapodius reinwardt)*	386.33	292.88	294.80	294.88	282.26	5	2
48	Pacific Baza *(Aviceda subcristata)*	98.21	97.61	94.28	96.19	96.24	3	3

AIC scores

	Species	Model 1 (flat)	Model 2 (monotonic)	Model 3 (plateau)	Model 4 (Gaussian)	Model 5 (skewed)	Top model	Top model ignoring skewness
49	Pied Imperial Pigeon *(Ducula bicolor)*	369.48	294.09	295.64	295.50	287.52	5	2
50	Pied Monarch *(Arses kaupi)*	533.45	516.87	492.30	510.41	494.29	3	3
51	Pale-yellow Robin *(Tregellasia capito)*	1074.22	939.10	875.96	901.36	877.15	3	3
52	Rainbow Bee-eater *(Merops ornatus)*	348.02	320.61	302.86	299.19	300.39	4	4
53	Rose-crowned Fruit-Dove *(Ptilinopus regina)*	141.78	122.51	124.51	124.51	126.51	2	2
54	Rufous Fantail *(Rhipidura rufifrons)*	537.14	490.09	492.09	492.09	494.09	2	2
55	Rainbow Lorikeet *(Trichoglossus haematodus)*	365.20	366.84	352.84	357.80	352.67	5	3
56	Red-necked Crake *(Rallina tricolor)*	76.47	71.91	73.22	72.81	73.39	2	2
57	Russet-tailed Thrush *(Zoothera heinei)*	54.31	56.27	53.10	55.24	46.07	5	3
58	Satin Bowerbird *(Ptilonorhynchus violaceus)*	461.63	363.58	359.85	360.52	358.53	5	3
59	Shining Bronze-Cuckoo (Golden) *(Chalcites lucidus)*	296.29	293.93	260.00	234.06	235.54	4	4
60	Scaly-breasted Lorikeet *(Trichoglossus chlorolepidotus)*	814.01	789.97	763.19	741.74	743.59	4	4
61	Scarlet Honeyeater *(Myzomela sanguinolenta)*	231.10	226.44	206.05	211.89	206.73	3	3
62	Sulphur-crested Cockatoo *(Cacatua galerita)*	363.45	361.78	341.91	340.56	337.77	5	4
63	Spangled Drongo *(Dicrurus bracteatus)*	201.34	187.55	189.55	189.55	191.55	2	2
64	Silvereye *(Zosterops lateralis)*	1081.42	928.12	839.95	861.44	839.67	5	3
65	Superb Fruit-Dove *(Ptilinopus superbus)*	381.86	371.91	363.09	367.77	364.90	3	3
66	Spectacled Monarch *(Symposiarchus trivirgatus)*	1048.86	878.41	860.42	864.94	839.60	5	3
67	Tooth-billed Bowerbird *(Scenopoeetes dentirostris)*	505.96	481.08	410.82	387.74	388.26	4	4
68	Topknot Pigeon *(Lopholaimus antarcticus)*	814.42	810.43	779.15	777.95	756.95	5	4
69	Victoria's Riflebird *(Ptiloris victoriae)*	340.39	332.97	307.91	298.88	300.03	4	4
70	Varied Triller *(Lalage leucomela)*	392.47	252.11	246.51	248.17	248.47	3	3
71	White-browed Robin *(Poecilodryas superciliosa)*	74.94	60.10	51.34	37.71	39.71	4	4
72	White-eared Monarch *(Carternornis leucotis)*	231.14	216.17	208.10	205.58	206.92	4	4

AIC scores

	Species	Model 1 (flat)	Model 2 (monotonic)	Model 3 (plateau)	Model 4 (Gaussian)	Model 5 (skewed)	Top model	Top model ignoring skewness
73	White-headed Pigeon *(Columba leucomela)*	263.75	265.71	267.71	267.71	269.71	1	1
74	Wompoo Fruit-Dove *(Ptilinopus magnificus)*	394.75	364.19	331.49	347.26	333.17	3	3
75	White-throated Treecreeper *(Cormobates leucophaea)*	544.17	483.21	410.42	420.08	403.07	5	3
76	Yellow-breasted Boatbill *(Machaerirhynchus flaviventer)*	588.79	505.34	471.01	472.58	470.05	5	3
77	Yellow-bellied Sunbird *(Nectarinia jugularis)*	283.86	191.88	176.69	190.92	178.67	3	3
78	Yellow Oriole *(Oriolus flavocinctus)*	157.99	129.87	112.64	114.24	105.19	5	3
79	Yellow-spotted Honeyeater *(Meliphaga notata)*	880.81	402.78	382.83	398.83	380.85	5	3
80	Yellow-throated Scrubwren *(Sericornis citreogularis)*	1710.21	1556.34	1356.82	1308.89	1293.38	5	4

Competing models in a hierarchical Huissman-Olff-Frescoe [30] model selection analysis amongst elevational density responses across the 88 Australian Wet Tropics rainforest bird species with sufficient sampling in this study are shown. Model selection was implemented using the approach implemented the R package “BiodiversityR” (Kindt 2011) (see methods for details).
